# Xenon Dynamics in Ionic Liquids: A Combined NMR and MD Simulation Study

**DOI:** 10.1021/acs.jpcb.0c03357

**Published:** 2020-07-02

**Authors:** Franca Castiglione, Giacomo Saielli, Michele Mauri, Roberto Simonutti, Andrea Mele

**Affiliations:** † Department of Chemistry, Materials and Chemical Engineering “G. Natta”, Politecnico di Milano, Piazza L. Da Vinci, 32, 20133 Milano, Italy; ‡ CNR—Istituto per la Tecnologia delle Membrane, Unità di Padova, Via Marzolo, 1, 35131 Padova, Italy; § Department of Chemical Sciences, University of Padova, Via Marzolo, 1, 35131 Padova, Italy; ∥ Dipartimento di Scienza dei Materiali, Università degli Studi di Milano Bicocca, Via Roberto Cozzi, 53, 20125 Milano, Italy; ⊥ CNR—SCITEC Istituto di Scienze e Tecnologie Chimiche, Via A. Corti 12, 20133 Milano, Italy

## Abstract

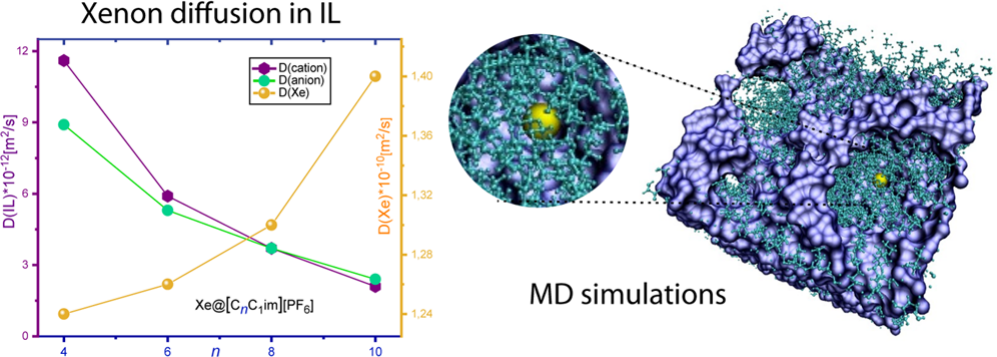

The translational dynamics of xenon gas dissolved in room-temperature ionic liquids (RTILs) is revealed by ^129^Xe NMR and molecular dynamics (MD) simulations. The dynamic behavior of xenon gas loaded in 1-alkyl-3-methylimidazolium chloride, [C*_n_*C_1_im]Cl (*n* = 6, 8, 10), and hexafluorophosphate, [C*_n_*C_1_im][PF_6_] (*n* = 4, 6, 8, 10) has been determined by measuring the ^129^Xe diffusion coefficients and NMR relaxation times. The analysis of the experimental NMR data demonstrates that, in these representative classes of ionic liquids, xenon motion is influenced by the length of the cation alkyl chain and anion type. ^129^Xe spin–lattice relaxation times are well described with a monoexponential function, indicating that xenon gas in ILs effectively experiences a single average environment. These experimental results can be rationalized based on the analysis of classical MD trajectories. The mechanism described here can be particularly useful in understanding the separation and adsorption properties of RTILs.

## Introduction

Room-temperature ionic liquids (RTILs) are a well-known class of materials characterized by a low melting point, low vapor pressure, and high chemical and thermal stability.^1−3^ Due to their peculiar physicochemical characteristics, IL solutions are ideal solvents for many reaction, separation, and extraction processes.^4−7^ Several studies have pointed out their utility in gas capture^8−10^ and separation, highlighting that the absorption capability strongly depends on the local liquid structure^11^ and mechanism of gas confinement.^12−14^

In light of these applications, a detailed understanding of the relationship between the IL local structure and the dynamic properties of gaseous species dissolved plays a central role and may help to design task-specific materials. Spectroscopy of noble gases,^15,16^ (especially xenon) loaded in nano/microstructured materials, has been used to probe the structure and the diffusion processes in porous media,^17^ zeolites,^18^ polymers,^19^ and nanochannels.^20^ Moreover, imaging NMR and diffusion measurements of thermally polarized and/or hyperpolarized xenon gas in free or confined spaces have been performed both at high and low magnetic fields^21,22^ and are also widely used for medical applications.^23−26^

Xenon gas, despite its chemical inertness, is particularly precious as an anesthetic in cardiovascular medicine and to treat drug addiction. Therefore, to improve its availability and reduce its cost, new MOF materials^27^ and zeolite membranes^28^ with tailored porosity have been proposed for selective xenon extraction and recycling. Among these new materials, the best adsorption and separation capacity is achieved when the pore size matches with the xenon kinetic diameter. Similarly, noble gas solubility^29^ in ILs strongly depends on the free volume that in turn is correlated with the nanostructure. The higher solubility of xenon compared with other nonpolar gases^30^ is due to its larger polarizability and subsequently, much stronger interactions with the IL.

**Scheme 1:**
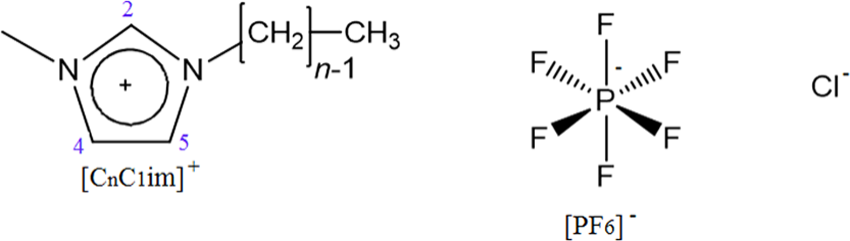
Molecular Structure of the Ionic Liquids Investigated in This Work (Imidazolium Ring Protons 2,4,5 Have Been Indicated)

In this article, we study the translational dynamics of xenon gas dissolved in some RTILs based on 1-alkyl-3-methylimidazolium chloride, [C*_n_*C_1_im]Cl (*n* = 6, 8, 10), and 1-alkyl-3-methylimidazolium hexafluorophosphate, [C*_n_*C_1_im][PF_6_] (*n* = 4, 6, 8, 10) (see Scheme 1 for molecular formulae). In particular, we consider the effect of the alkyl chain length of the IL cation on the motion regime of xenon gas. The diffusivity of xenon atoms, IL cations, and anions was independently examined by means of multinuclear pulsed gradient spin echo (PGSE) ^129^Xe, ^1^H, and ^19^F NMR spectroscopy. Variable diffusion time experiments^31^ allowed us to study the diffusion motion in the time range of milliseconds to seconds. Moreover, xenon spin–lattice relaxation times *T*_1_ are measured using ^129^Xe inversion recovery experiments to evaluate atomic dynamics in the picosecond timescale. The experimental data were analyzed following a conventional methodology suitable to evaluate free or restricted motion in liquids and in the gel phase. Finally, the dynamics of xenon gas loaded in *n*-alkanes [C*_n_*H_2*n*+2_] *n* = 6, 8,10, liquid at room temperature, is also investigated, and the results of the pure liquids *vs* RTIL are compared. Our approach provides the characterization and the comparison of Xe@RTILs and Xe@alkanes’ dynamics for the first time.

In parallel, classical molecular dynamics (MD) simulations of Xe@[C*_n_*C_1_im][X], where X = Cl,^–^ PF_6_^–^ (*n* = 2, 4, 6, 8, 10), and Xe@alkanes (*n* = 6, 10) are also carried out. The outcome of the simulations is compared with experimental results obtained via NMR spectroscopy.

## Experimental Methods

### Materials

All ionic liquids and alkanes were acquired from Aldrich and used without further purification. The chemical structures of all ions are depicted in Scheme 1.

### NMR Sample Preparation

NMR “medium wall” tubes with a 5 mm external diameter and a 3.46 mm internal diameter were acquired from Wilmad. The tubes were filled roughly to the same height (5 cm) with ionic liquids and a short/thin capillary tube containing DMSO-*d*_6_ (60–100 μL) was manually inserted (Figure S3). The samples were dehydrated overnight at 343 K under dynamic vacuum (mechanical pump, usually less than 20 Pa, i.e., 1.4 × 10^–1^ Torr). Afterward, the tubes were connected to a vacuum system and degassed several times by freeze–thaw technique and less than 8 Pa pressure (6 × 10^–2^ Torr). Xenon gas was initially contained in a known reservoir volume (28.29 mL), with an initial pressure of 150 Torr (20 kPa). The volume was then put in contact with the NMR tube using Wilmad connectors. The volume of these necessary connectors (15 mL) was measured prior to sample preparation by nitrogen gas expansion using the same tubes. Xenon gas was then frozen in the tube using liquid nitrogen, and the tube was flame-sealed. Then, the sample was let to equilibrate for a week. The final nominal pressure of xenon gas was around 3.5 atom for all of the samples (see the Supporting Information for details and Figures S1–S3). Xenon solubility in ILs has been investigated in a wide range of temperature and up to 0.3 MPa pressure.^29^

### NMR Spectroscopy

^129^Xe NMR experiments were carried out on a Bruker DRX 500 spectrometer equipped with a 5 mm broadband inverse probehead. The ^129^Xe PGSE experiments were performed using a stimulated-echo pulse sequence. The acquisition parameters were δ = 3 ms and Δ = (*t*_d_ – δ/3) = 0.5–2 s in a step of 0.1 s for Xe@C_10_C_1_imCl for variable observation time measurements and δ = 3 ms and Δ = 0.72 s for all of the other IL samples. A relaxation delay, *D*_1_, of 40–80 s and 80 acquisition scans were used for all of the experiments for a total acquisition time of 15–30 h each. *T*_1_ relaxation times of ^129^Xe were measured using inversion recovery pulse sequence. The recycling delays were in the range 0.5–120 s and the number of accumulated scans was 66 for all samples. The acquisition parameters for diffusion experiments on alkane samples were *D*_1_ = 900 s, δ = 3 ms, and Δ = 0.032 s. For relaxation experiments, the recycling delays were in the range 20–2600 s and 8 acquisition scans.

^1^H and ^19^F experiments were performed on a Bruker AVANCE spectrometer operating at 500.13 MHz proton frequency equipped with a four nuclei switchable probe (QNP). PGSE data were acquired using the bipolar pulse-longitudinal eddy current delay (BPP-LED) pulse sequence with the following parameters: 16 scans; relaxation delay of 10 s; δ = 3 ms and Δ = 1 s for IL samples; and δ = 2 ms and Δ = 0.02 s for alkane samples. A pulsed gradient unit capable of producing magnetic field pulse gradients in the *z* direction of 53 G/cm was used. The temperature was set and controlled at 305 K.

### MD Simulations

We have used the software package Gromacs^32^ to run MD simulations of several IL systems. The force field (FF) used features the charge distribution developed by Canongia-Lopes and Padua (CL&AP FF),^33^ while the internal parameters are based on the Amber^34^ FF implementation in Gromacs. All bonds were constrained by the LINCS algorithm.^35^ The leap-frog integrator was used with a time step of 1 fs and a cutoff of 10 Å for the van der Waals and short-range electrostatic interaction. The particle–mesh Ewald (PME)^36^ technique was used to handle long-range electrostatic interactions with an interpolation order of 4. Simulations were run in the NPT ensemble using the Berendsen thermostat^37^ and the Parrinello–Rahman barostat^38,39^ with applied isotropic periodic boundary conditions.

Boxes were built starting from previous simulations^40,41^ of the butyl systems, changing gradually the alkyl chain length after expanding the box to avoid overlap. Each system was then quickly relaxed to the volume under NPT conditions and equilibrated for 12 ns. The equilibration run was followed by a production run of 60 ns; configurations were saved every picosecond for further analysis. A first set of simulations was run with a box containing 500 ion pairs of [C*_n_*C_1_im][X] (*n* = 2, 4, 6, 8, 10 and X = Cl^–^, PF_6_^–^) plus a Xe atom; for all systems, these boxes were simulated at 350, 400, 450, and 500 K and pressure of 1 bar. Inspection of the cation and anion diffusion coefficients revealed that some short-chain systems at the lower temperature were in a glassy state rather than a liquid state. In a second set of simulations, the temperature was set to 400 K and the pressure to 1 bar. The boxes of this second set contained 250 ion pairs plus a Xe atom. Three independent runs were produced for each system to estimate, together with the results of the first set of simulation at the same temperature of 400 K, the error associated with the diffusion coefficient of xenon. The systems studied by MD simulations are reported in Table S1.

Additional simulations were run for Xe@hexane and Xe@decane to estimate the diffusion of xenon in the liquid alkane. The FF parameters for the two alkanes were the same used for the hydrophobic part of the alkyl chain of the imidazolium salts. Again, three independent boxes were generated containing 250 alkane molecules and one xenon atom. The boxes were equilibrated for 30 ns and the subsequent three consecutive production runs lasted 60 ns. Since there is no significant effect of electrostatic interaction for these systems, as for ILs, slowing down the dynamics, we ran the simulations at 300 K.

The Gromacs built-in software utilities were used to calculate radial distribution functions (RDFs) and mean-squared displacements (MSDs). The diffusion coefficient was then obtained by linear fitting of the MSD in an appropriate range: for the cation and anion diffusion coefficients, the MSD was found to be linear normally up to 50 ns; for the single Xe atom, we limited the fitting to the first 2 ns. The first 0.2 ns were excluded from the linear fitting procedure.

## Results and Discussion

### NMR Diffusion

NMR diffusion experiments^42^ are based on the measurement of the signal decay after applying a train of field gradient pulses (PFG) of duration δ and increasing intensity *g* along the *z* direction. The signal decay intensity *I*(*q*, *t*_d_) measured at a fixed time *t*_d_ can be related to the mean-squared displacement (MSD), ⟨*z*^2^⟩, as follows
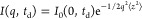
1where *q* = (δγ*g*)/2π and γ is the magnetogyric ratio of the observed nucleus. In the case of diffusing species whose motion is described by the Langevin^43^ equation (hence Fickian diffusion), the MSD scales linearly with the observation time *t*_d_ according to eq 2 obtained for the case of application of field gradients along the *z* direction only
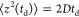
2with *D* being the particle self-diffusion coefficient. This relation properly describes not only the free diffusion motion of liquid samples but also all of the diffusion processes that, even in the presence of barriers or obstacles, are described by a Gaussian distribution of displacement probabilities.^44,45^ This condition occurs whenever the observation time *t*_d_ and the mean diffusion distance ⟨*z*⟩ = (MSD)^1/2^ traveled by the molecules during *t*_d_ become much larger than the characteristic length-scales λ associated with the obstacles.^46^

Different from free diffusion, in complex heterogeneous systems, whenever ⟨*z*⟩ ∼ λ the molecule feels the effects of the obstacles and the MSD is related to the elapsed time *t*_d_ through a more general equation
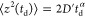
3where *D*′ is a generalized diffusion coefficient (whose units are α-dependent) and the parameter α ≠ 1 is defined as the anomalous diffusion exponent. The motion regime may be defined as non-Fickian and, depending on the α value, as anomalous subdiffusive (0 < α < 1) or anomalous superdiffusive (α > 1). Only a few systems deviate from this equation, such as molecular crystals,^20,47^ where geometrical constraints to the motion produce a non-Gaussian distribution of displacements.

**Figure 1:**
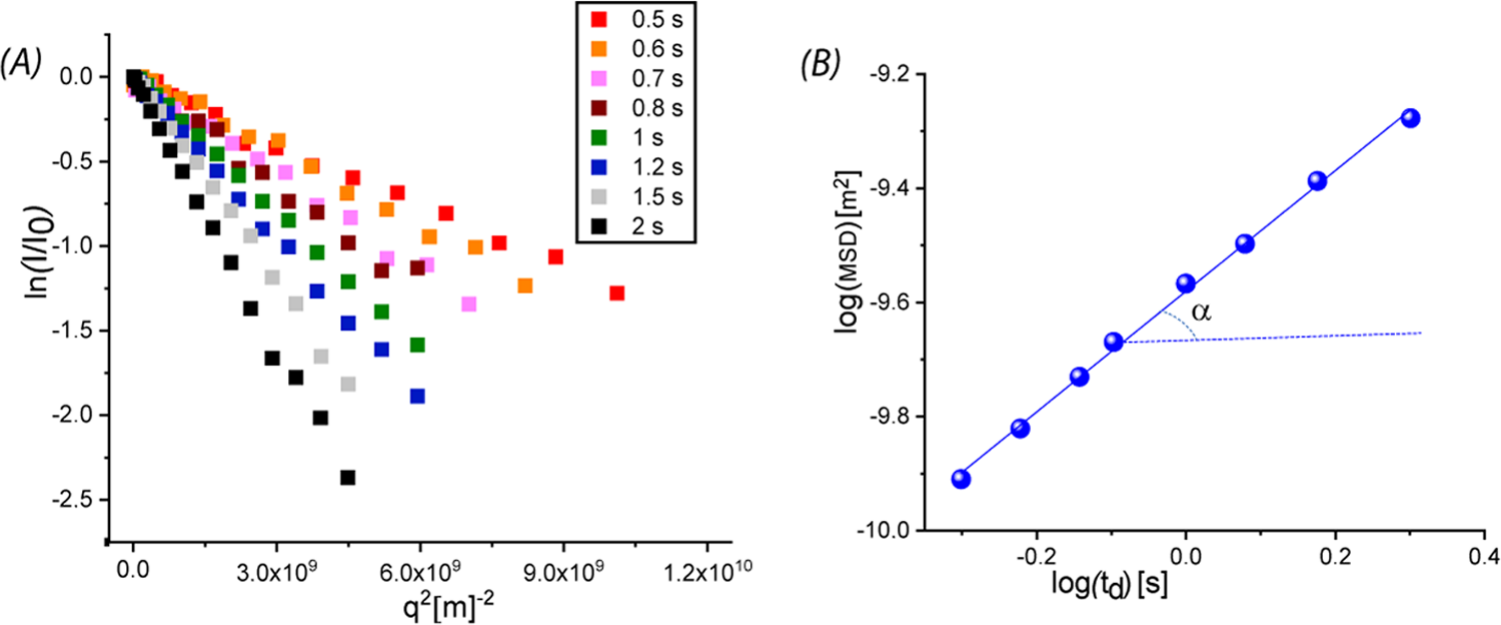
(A) Normalized NMR signal decay plotted on a semilogarithmic scale *vs q*^2^ for Xe@[C_10_C_1_im]Cl. (B) Log–log plot of mean-squared displacements as a function of the observation time *t*_d_. Line fitting of the experimental data is also reported. All of the experiments were carried out at 305 K.

Figure 1A shows the normalized experimental signal decay *I*(*q*, *t*_d_) plotted on a semilogarithmic scale *vs q*^2^ for Xe@C_10_C_1_imCl with observation time *t*_d_ in the range 0.5–2 s. The slopes of the linear fits provide the MSD values for each observation time. The log–log plot of xenon MSD *vs t*_d_ is also reported in Figure 1B. It is important to note that a log–log plot based on eq 3 provides the experimental α values as the slope and *D*′ as the intercept of the linear regression. An immediate indication of the motion regime is obtained.

The analysis of xenon motion was performed for Xe@[C_10_C_1_im]Cl sample and the scaling exponent α was found to be 1.05 ± 0.02, providing evidence of Fickian diffusion for the gas in an IL environment. This indicates that the diffusing Xe atoms undergo unrestricted diffusion and do not experience diffusion barriers or obstacles of length-scale comparable with ⟨*z*⟩ (12–24 μm) accessible by PGSE experiments during the observation time (0.5–2 s). The diffusion coefficients can be calculated according to eq 2 and they are reported in Table 1 for Xe@[C*_n_*C_1_im]Cl and Xe@[C*_n_*C_1_im][PF_6_]. In parallel, the diffusion coefficients of the IL cation and hexafluorophosphate anion were also measured using ^1^H and ^19^F NMR. A summary of the experimental diffusion coefficients is reported in Table 1. Table 1 also collects the *T*_1_ data, which will be discussed in the next section.

**Table 1:** PGSE-NMR Diffusion Coefficients of Xenon@ILs (*D*_Xe_), IL Cations *D*(^1^H), and Anions *D*(^19^F) for Xe@[C*_n_*C_1_im]Cl and Xe@[C*_n_*C_1_im][PF_6_] samples. Diffusion coefficients of xenon@alkanes (*D*_Xe_) and alkanes *D*(^1^H) for Xe@[C*_n_*H_2*n*+2_] samples. *T*_1_relaxation time of xenon gas loaded in ILs and alkanes.^a^

samples	*D*(Xe) (m^2^/s)	*D*(^1^H) (m^2^/s)	*D*(^19^F) (m^2^/s)	*T*_1_(Xe) (s)
Xe@[C_6_C_1_im][Cl]	0.63 × 10^–10^	2.9 × 10^–12^		7.3
Xe@[C_8_C_1_im][Cl]	0.77 × 10^–10^	0.5 × 10^–12^		12.2
Xe@[C_10_C_1_im][Cl]	1.45 × 10^–10^	0.5 × 10^–12^		16.1
Xe@[C_4_C_1_im][PF_6_]	1.24 × 10^–10^	11.6 × 10^–12^	8.9 × 10^–12^	13.9
Xe@[C_6_C_1_im][PF_6_]	1.26 × 10^–10^	5.9 × 10^–12^	5.3 × 10^–12^	13.8
Xe@[C_8_C_1_im][PF_6_]	1.30 × 10^–10^	3.7 × 10^–12^	3.7 × 10^–12^	15.8
Xe@[C_10_C_1_im][PF_6_]	1.41 × 10^–10^	2.1 × 10^–12^	2.4 × 10^–12^	18.1
Xe@[C_6_H_14_]	7.66 × 10^–9^	5.0 × 10^–9^		217.0
Xe@[C_8_H_18_]	5.51 × 10^–9^	2.8 × 10^–9^		203.9
Xe@[C_10_H_22_]	5.05 × 10^–9^	1.2 × 10^–9^		170.9

a All of the data were obtained at 305 K. The experimental data are estimated to be accurate between ±3 and ±6%.

Xenon diffusion in ILs is many orders of magnitude smaller than that of free xenon gas^48^ (5.3 × 10^–6^ m^2^/s) and about 1 order of magnitude smaller than xenon dissolved in water^49^ (2.2 × 10^–9^ m^2^/s) or alkanes, thereby indicating that xenon dynamics is influenced by the peculiar structural features of the IL systems. Furthermore, xenon diffusion is about 2 orders of magnitude faster than the diffusion of both the IL’s cation and anion. Thus, the dynamics of the observed species follows the general trend *D*(Xe) ≫ *D*(cat) ≥ *D*(an) for all of the samples, despite the different anion and chain lengths. This finding also pointed out that the diffusivity of Xe is scarcely influenced by the different viscosities of the alkylimidazolium ILs as a function of the length of the alkyl chain.

### ^129^Xe NMR Relaxation

Spin–lattice relaxation^50^ designated by the time constant *T*_1_ is sensitive to the magnetic intra/intermolecular interactions as well as to their time dependence arising from molecular tumbling in solution. Among the several relaxation mechanisms, spin–rotation interaction is responsible for xenon relaxation in the gas phase, while ^129^Xe–^1^H dipole–dipole coupling is the predominant mechanism accounting for xenon relaxation in solution.^51^^129^Xe *T*_1_ values, reported in Table 1, are obtained by fitting the experimental data with a monoexponential function, suggesting that xenon gas in all ILs, as well as in alkane samples, experiences a single average environment.

**Figure 2:**
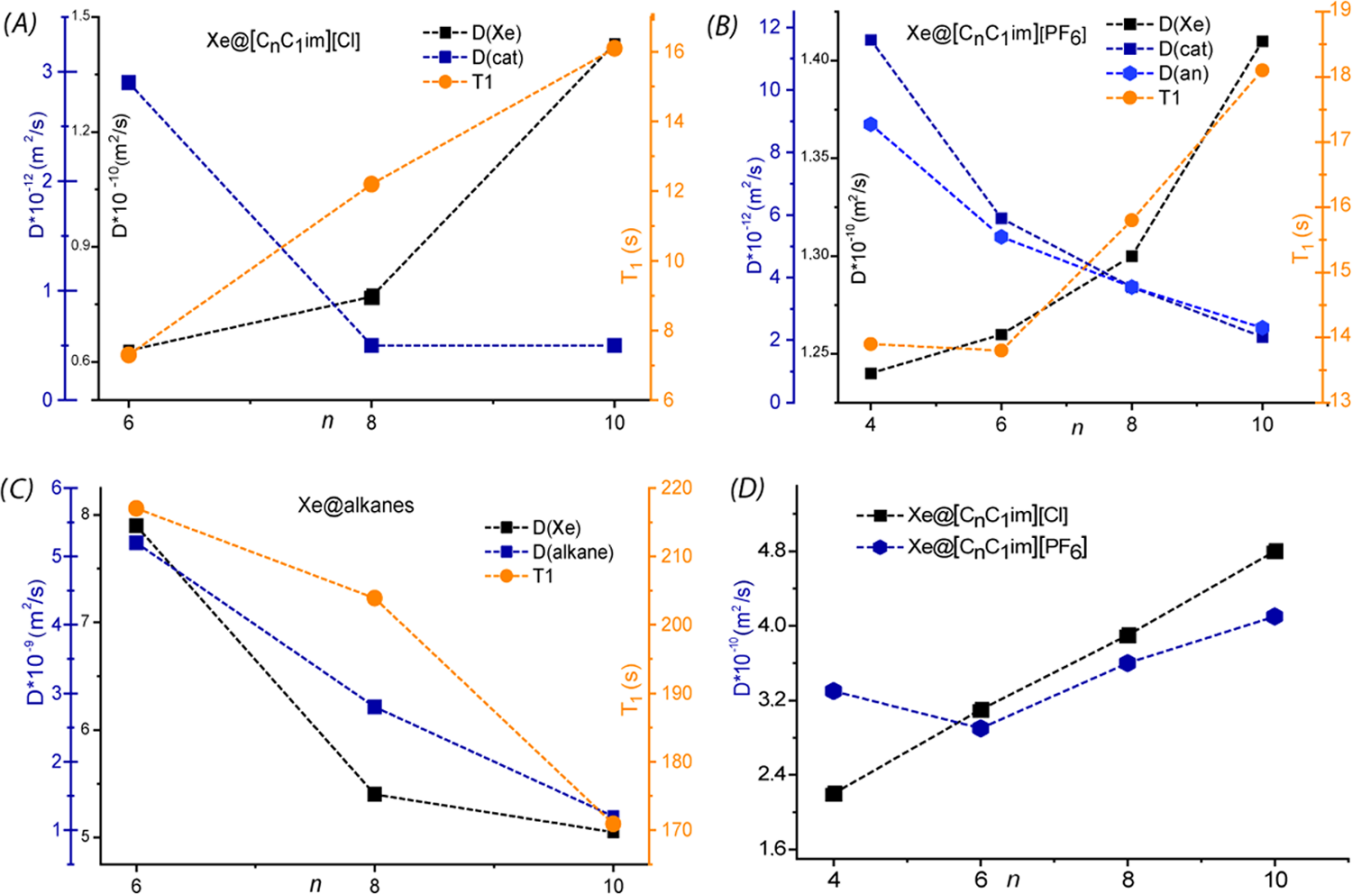
(A–C) Plot of the experimental diffusion coefficients *D*(Xe), *D*(cat), *D*(an), and xenon *T*_1_*vs* alkyl chain length, *n*, for all ILs and alkane samples. (D) Plot of *D*(Xe) calculated with MD simulation for the two IL samples. A system of double *y* axis has been used for plots (A)–(C) along with a color code. For better clarity, in plot (A) the blue *y* axis reports the diffusivity scale for the imidazolium cation of the ILs, in turn reported as blue squares. In a similar way, the black *y* axis reports the scale for *D*(Xe) dissolved in the same ILs. The secondary *y* axis (orange) on the right-hand side of the plot reports the *T*_1_ scale in seconds, while the experimental *T*_1_ are the orange squares in the plots. The same legend holds for plot (B) and (C) for Xe@alkanes. The two left *y* axis in graph (C) have the same scale.

Different dynamic behavior of xenon is observed in the two sets of IL and alkane samples; the results, reported in Table 1 and Figure 2, can be summarized as follows. (i) In alkane samples, *D*(Xe), xenon relaxation time *T*_1_ and *D*(C*_n_*H_2*n*+2_) decrease on increasing the alkyl chain length (Figure 2C) according to an increase of sample viscosity. (ii) In chloride-based IL, *D*(Xe) and relaxation time *T*_1_ increase with alkyl chain length on going from Xe@[C_6_C_1_im]Cl to Xe@[C10imC_1_]Cl (Figure 2A). (iii) A similar, but nonidentical, behavior is observed in Xe@[C*_n_*C_1_im][PF_6_] samples: the *D*(Xe) values remain almost unchanged on passing from butyl to decyl alkyl chains, whereas Xe *T*_1_ values increase in the same order (Figure 2B). (iv) For all samples, the measured Xe diffusivity shows an opposite trend with respect to the cation/anion of the ionic liquid on passing from small to large *n* values (i.e., with progressively longer alkyl chains): while the components of the ILs diffuse at a slower rate with larger *n*, Xe diffusivity grows correspondingly. The latter finding is unexpected and counterintuitive, indicating that the two motional regimes are decoupled. Actually, the available literature data on the viscosity of the ILs under investigation, taken from the database published by Yu et al.,^52^ clearly indicate that the viscosity increases with the increasing length of the alkyl chains for both the PF_6_^–^ and Cl^–^ series at the same conditions of *T* and *P*. The *D* values of Table 1 related to both cations and anions of the examined ILs decrease with increasing viscosity, whereas the corresponding *D*(Xe) and *T*_1_ relaxation values are not affected by the solvent viscosity in the same way. The transport of Xe atoms in the ILs seems to be related to the extension of the nonpolar domain, as indicated by the extension of *n*, and basically independent of the motion of the anion–cation components of the ILs.

To better understand the structural features responsible for the differences experimentally observed in Xe@IL motion, we performed classical MD simulations as described below (see the Supporting Information for additional details). Since the electrostatic interaction in nonpolarizable force fields is known to significantly slow down the dynamics and the sampling of the phase space,^53^ we used a higher temperature compared to experiments for Xe@IL systems, *T* = 400 K. The results of these simulations, therefore, only have a qualitative meaning but nonetheless, as we will see, they provide essential insights into the interpretation of the results.

### MD Simulations

The results of classical MD simulations are reported in Table 2 and Figure 2D.

**Table 2:** MD Simulation Diffusion Coefficients of Xenon *D*(Xe), Ionic Liquid Cations *D*(cat), and Anion *D*(an) for Xe@[C*_n_*C_1_im]Cl and Xe@[C*_n_*C_1_im][PF_6_] Samples at *T* = 400 K and of Xenon and Alkanes for Xe@hexane and Xe@decane at *T* = 300 K^a^

sample	*D*(Xe) (m^2^/s)	*D*(cat) (m^2^/s)	*D*(an) (m^2^/s)
Xe@[C_2_C_1_im][Cl]	2.5 × 10^–10^	5.2 × 10^–11^	3.4 × 10^–11^
Xe@[C_4_C_1_im][Cl]	2.2 × 10^–10^	2.2 × 10^–11^	2.0 × 10^–11^
Xe@[C_6_C_1_im][Cl]	3.1 × 10^–10^	7.9 × 10^–12^	8.0 × 10^–12^
Xe@[C_8_C_1_im][Cl]	3.9 × 10^–10^	4.3 × 10^–12^	5.0 × 10^–12^
Xe@[C_10_C_1_im][Cl]	4.8 × 10^–10^	2.7 × 10^–12^	3.2 × 10^–12^
Xe@[C_2_C_1_im][PF_6_]	3.6 × 10^–10^	5.2 × 10^–11^	2.9 × 10^–11^
Xe@[C_4_C_1_im][PF_6_]	3.3 × 10^–10^	3.7 × 10^–11^	2.2 × 10^–11^
Xe@[C_6_C_1_im][PF_6_]	2.9 × 10^–10^	1.8 × 10^–11^	1.4 × 10^–11^
Xe@[C_8_C_1_im][PF_6_]	3.6 × 10^–10^	1.0 × 10^–11^	9.3 × 10^–12^
Xe@[C_10_C_1_im][PF_6_]	4.1 × 10^–10^	6.6 × 10^–12^	6.5 × 10^–12^
Xe@[C_6_H_14_]^b^	5.0 × 10^–9^	2.7 × 10^–9^^c^	
Xe@[C_10_H_22_]^b^	1.5 × 10^–9^	6.5 × 10^–10^^c^	

a Errors are estimated as the average absolute deviation over three independent runs. They are between ±1 and ±5% for the diffusion coefficients of the ions and about ±10% for the diffusion coefficient of xenon.

b *T* = 300 K.

c *D*(alkane).

First, the results for the two simulated systems Xe@alkanes compare very well with the experiments. We note that, being noncharged systems, nonpolarizable force fields are expected to perform rather well, almost at a quantitative level; see Tables 1 and 2. Concerning the ionic systems, in Figure S11, we see an analogous trend to the experimental data of the diffusion coefficients of cations and anions as the chain length is increased; moreover, the cation of the chloride salt has a slower diffusion than the cation of the hexafluorophosphate salt for the same alkyl chain length, both in experiments and simulations, confirming the reliability of the simulations of the Xe@IL systems at least from a qualitative point of view.

For Xe@[C*_n_*C_1_im][PF_6_], *D*(Xe) varies relatively little from the system C_2_ (3.6 × 10^–10^ m^2^/s) to the system C_10_ (4.1 × 10^–10^ m^2^/s), though it appears to have a minimum variation for the C_6_ salt. In contrast, for the chloride salt, the xenon diffusion is much more strongly dependent on the chain length, as observed experimentally. Moreover, the diffusion of xenon in the hexafluorophosphate salt is faster than in the chloride salt for short chains, while the two diffusion coefficients tend to become closer for longer chains. It is also worth to mention that the Xe dynamics described by the MD simulations appears to be well described by a linear dependence of the MSD with time; see Figures S8–S10 in the Supporting Information.

**Figure 3:**
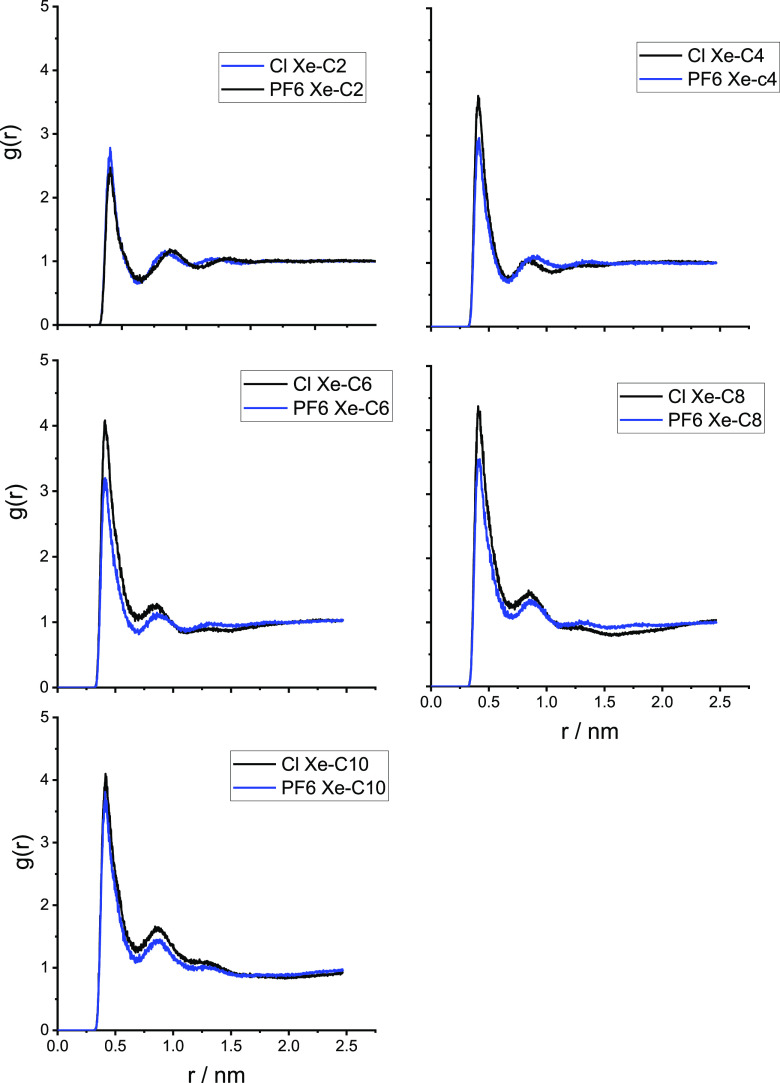
Radial distribution functions of the distance between Xe and the terminal methyl group of the alkyl chain [C*_n_*C_1_im]Cl (black) and [C*_n_*C_1_im][PF_6_] (blue).

**Figure 4:**
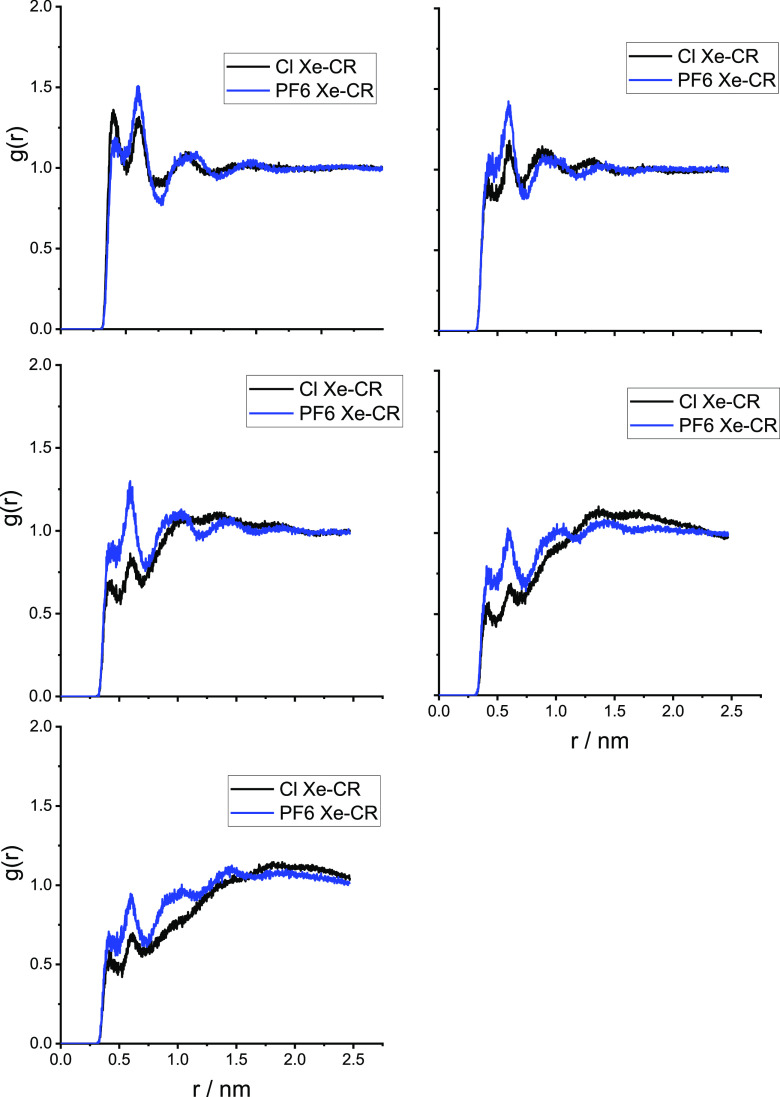
Radial distribution functions of the distance between Xe and carbon C2 (see Scheme 1) of the imidazolium ring labeled CR. [C*_n_*C_1_im]Cl (black) and [C*_n_*C_1_im][PF_6_] (blue).

**Figure 5:**
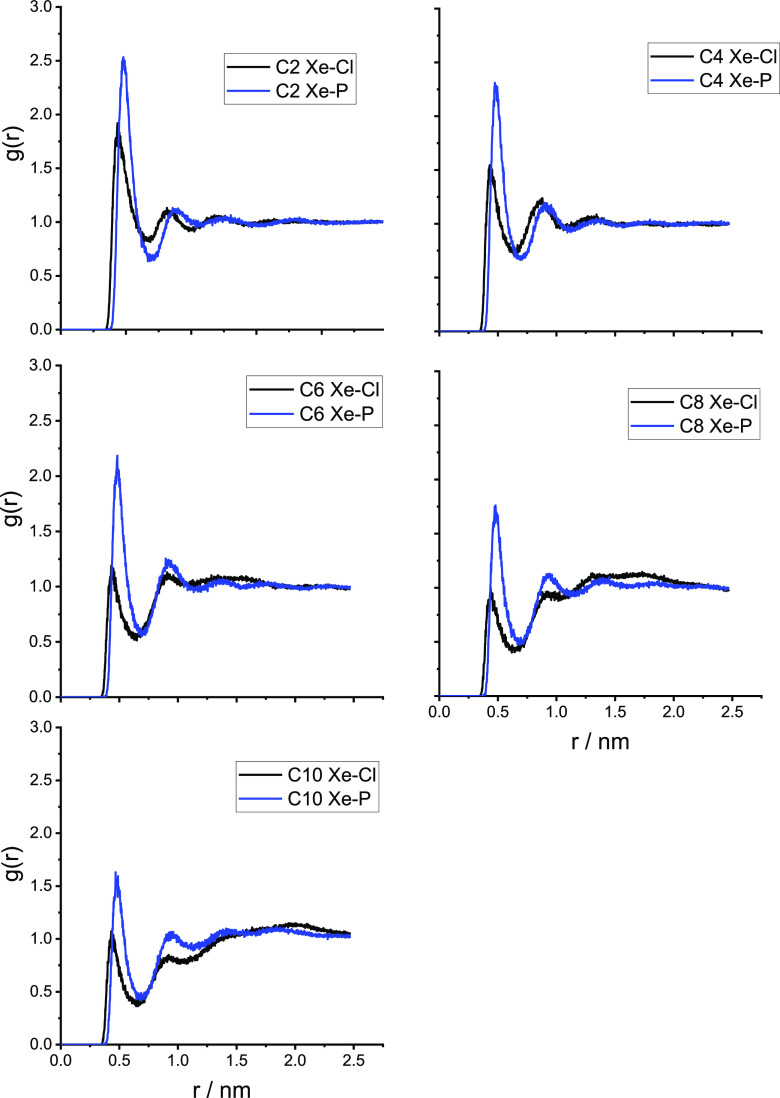
Radial distribution functions of the distance between Xe and the anion. [C*_n_*C_1_im]Cl (black) and [C*_n_*C_1_im][PF_6_] (blue).

It is possible to interpret these data by considering the structural features of the two systems, as obtained from MD simulations and previously validated by a comparison of experimental and calculated xenon chemical shifts.^40^ It is well known that in ILs, the ionic parts are, on average, arranged in a continuum polar network separated by the hydrophobic domains.^54−56^ In Figures 3–5, we show the radial distribution functions (RDFs or *g*(*r*)) of Xe with some selected atoms of the ionic liquids: these are the terminal methyl carbons of the alkyl chain in Figure 3; the imidazolium ring carbon in position 2 of the ring (see Scheme 1), labeled CR in Figure 4; and the anion in Figure 5. The RDFs clearly show that xenon is preferentially solvated by the alkyl chains rather than by the ionic moieties of the IL (see also ref (57)), as indicated by the first strong peak in the RDFs in Figure 3; moreover, for the chloride salts, such solvation appears stronger than with the corresponding hexafluorophosphate salts. From the RDFs in Figure 4, it is clear that the interaction of Xe with the imidazolium ring is very weak in all cases. However, the hydrophobic anion [PF_6_^–^] can penetrate into the hydrophobic alkyl domains more easily than the hard and hydrophilic Cl^–^. This is evident from Figure 5 where the peak in the RDF of Xe with the P atom in the [PF_6_] salt is significantly higher in intensity than the analogous one with chloride. Therefore, the nanosegregation is stronger and more defined in the chloride salt compared to the hexafluorophosphate salt. As the chain length increases, the hydrophobic domains become larger but also more connected in the chloride salt.

**Figure 6:**
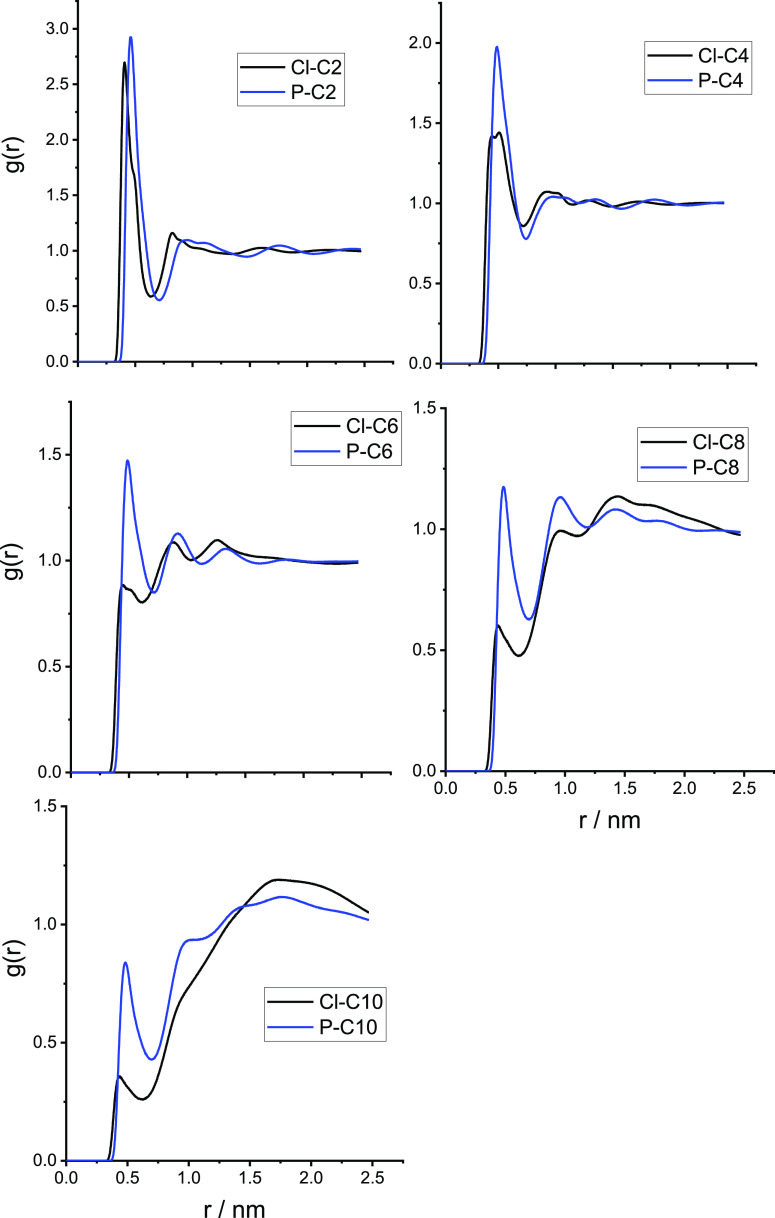
Radial distribution functions of the distance between the terminal methyl group of the alkyl chain and the anion center of mass for [C*_n_*C_1_im]Cl (black) and [C*_n_*C_1_im][PF_6_] (blue).

Finally, the RDF between the center of mass of the anion and the terminal methyl group of the chain, see Figure 6, shows a clear peak in the probability of finding the hexafluorophosphate at contact distance with the terminal methyl group even for the C10 systems, while such probability is strongly reduced for the chloride salt. This confirms that PF_6_^–^ can, to some extent, penetrate the hydrophobic domains where xenon is preferentially solvated, in agreement with ref (40) (see also Figure S12 in the Supporting Information).

**Figure 7:**
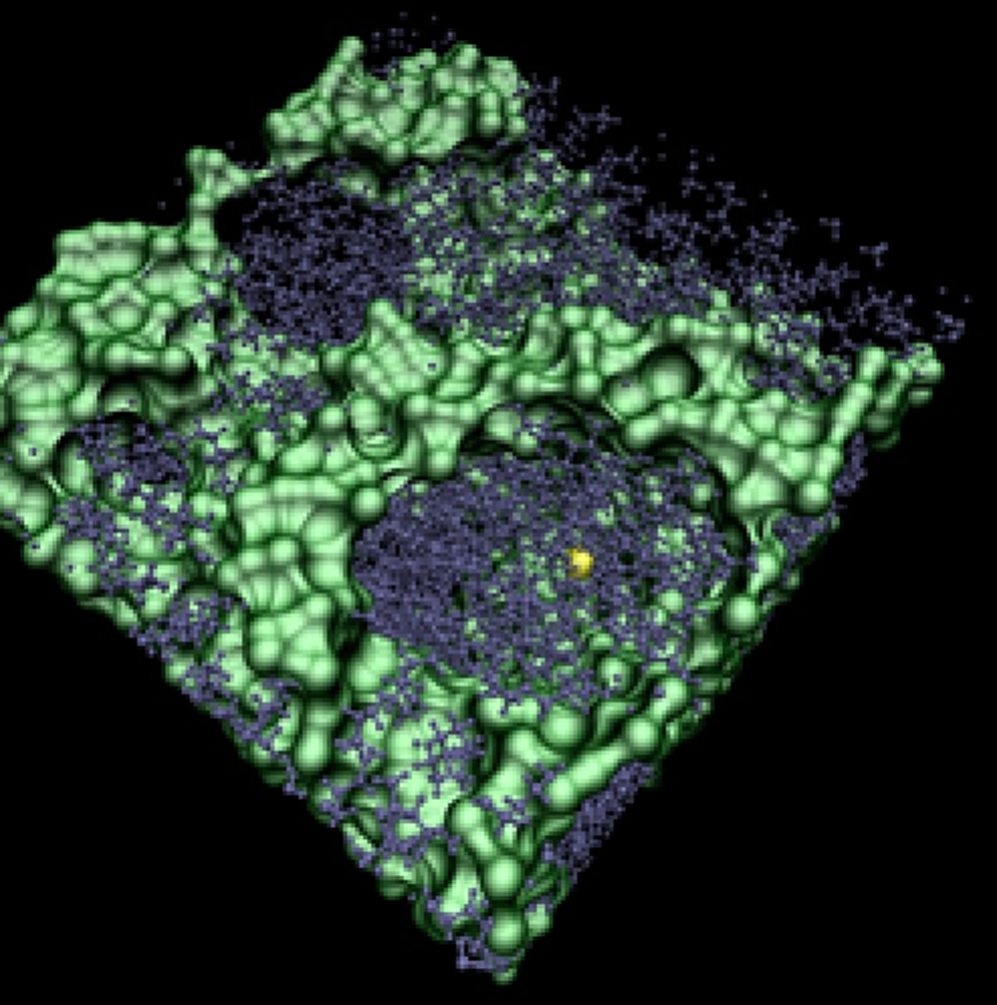
Snapshot of Xe@[C_10_C_1_im]Cl showing the Xe atom, in yellow, within a nanosegregated alkyl domain (dark gray). The ionic network of imidazolium heads and chloride anions is rendered as a connected smoothed volume in light green. Graphical rendering with visual molecular dynamics (VMD).^58^

This means that for the chloride salt, increasing the alkyl chain length produces a significant change in the environment felt by xenon, that is, the growing hydrophobic domain that become more and more segregated from the polar network of ions; in contrast, for the hexafluorophosphate salt, such a change is to some extent mitigated by the fact that the anions are more easily dispersed within the hydrophobic domain and such a domain is, in fact, more loosely defined than for the chloride salt. Therefore, the smoother the change in the environment as the chain length is increased, the weaker the dependence of the diffusion coefficient, as observed in Figure 2. Besides the differences between the two systems, however, why does the *D*(Xe) increase with the chain length, especially for the chloride salt? We believe this to be a result of larger hydrophobic domains, with a corresponding increased fraction of free volume being available for diffusion, which become more and more interconnected, thus offering a way to increase the Xe translational motion. Both trends of the Xe diffusion coefficients (from experimental NMR and MD simulations) and of Xe relaxation time *T*_1_ support the picture of an interconnected network of hydrophobic domains where xenon preferentially resides and diffuses. Their dependence on the chain length reflects the extent of the variation of the structural features of such domains in the studied ILs, see Figure 7.

The values of *D*(Xe) and *T*_1_ in the chloride and hexafluorophosphate salts are quite different for short chains, while they tend to become closer for longer alkyl chains. These results are also in agreement with previous data on ^129^Xe chemical shift in the same ILs.^57,59^ δ(^129^Xe) was found to be highly dependent on chain length although the nature of the anion can invert the slope of the variation: in chloride-based ILs, δ(^129^Xe) decreases with increasing alkyl side-chain lengths, meanwhile for the series based on [PF_6_] ion, xenon chemical shift increases. In both cases, δ(^129^Xe) to converge to a common value for long-chain ILs as the hydrophobic domains, preferentially hosting the Xe atom, becomes more relevant.

## Conclusions

Combined NMR diffusion-relaxation experimental data and computational MD simulation of ^129^Xe gas in two representative classes of IL systems provided relevant information on the structure–dynamics relationship. The measured diffusivity for Xe@[C_10_C_1_im]Cl exhibits a linear relation with the observation time (Fickian diffusion). This indicates that in the IL nanostructure, at least for this system with relatively long alkyl chains, there are no diffusion barriers, and xenon atoms diffuse in a more rigid homogeneous medium. This picture is confirmed by the results of the MD simulations that show an interconnected network of alkyl domains. Nevertheless, the alkyl chain length and type of anion, and hence the detailed structure of the nanosegregated domains, influence the gas diffusion coefficient and spin–lattice relaxation. This can be particularly appreciated by a comparison of the dynamics in simple alkanes: here, the Xe diffusion, alkane diffusion, and ^129^Xe *T*_1_ decrease with increasing chain length because of the increasing viscosity. For xenon, the opposite trend is observed in ILs, that is, an increase of the diffusive motion and *T*_1_ with increasing chain length, while the cations and anions still exhibit the expected trend with viscosity.

These results improve significantly the understanding of noble gases’ motion in innovative materials such as RTILs, thus facilitating their use for cost-efficient Xe recycling and recovery as well as other conceivable industrial applications.

## Supporting Information Available

Experimental details for Xe@IL sample preparation and processing of the ^129^Xe NMR data; details on the molecular dynamics procedures (PDF)
