# Supplementary material for: Xenon Dynamics in Ionic Liquids: A Combined NMR and MD Simulation Study
Source: J Phys Chem B. 2020 Jul 2;124(30):6617–27. doi: 10.1021/acs.jpcb.0c03357 (PMC8009510; doi:10.1021/acs.jpcb.0c03357)
Supplement: Supplementary file 1 — jp0c03357_si_001.pdf [file jp0c03357_si_001.pdf]

Supporting Information

Xenon dynamics in Ionic Liquids: a combined NMR and MD simulations study

Franca Castiglione,[a] Giacomo Saielli,[b,c]* Michele Mauri,[d] Roberto Simonutti,[d] Andrea Mele[a,e]*

[a]Department of Chemistry, Materials and Chemical Engineering "G. Natta", Politecnico di Milano, Piazza L. Da Vinci, 32, 20133 Milano, Italy.

[b]CNR – Istituto per la Tecnologia delle Membrane, Unità di Padova, Via Marzolo, 1 – 35131 Padova, Italy.

[c]Department of Chemical Sciences, University of Padova, Via Marzolo, 1 – 35131 Padova, Italy.

[d]Dipartimento di Scienza dei Materiali, Università degli Studi di Milano Bicocca, Via Roberto Cozzi, 53, 20125 Milano, Italy.

[e]CNR – SCITEC Istituto di Scienze e Tecnologie Chimiche, Via A. Corti 12, 20133 Milano, Italy. Italy.

S.1 Experimental

Xenon Loading Experimental Setup

We used Xe 5.0 gas at natural abundance. The final pressure of xenon gas in the NMR tube is calculated considering the tube internal diameter, the volume and initial pressure of the reservoir, the total volume of the cross + connector + tube system and the final residual pressure after gas solidification. The pressure is nominal since it is calculated from the volume of the empty tube and thus does not consider

1) The volume occupied by the capillary, which is necessary for instrument lock
2) The solubility of xenon in the IL
3) Some volume reduction due to the sealing.

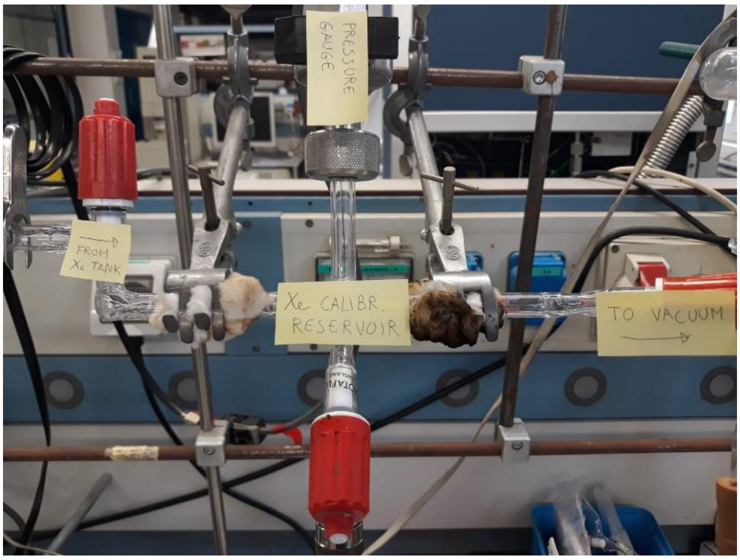

Figure SI1. View of the apparatus used for preparing Xe NMR samples.

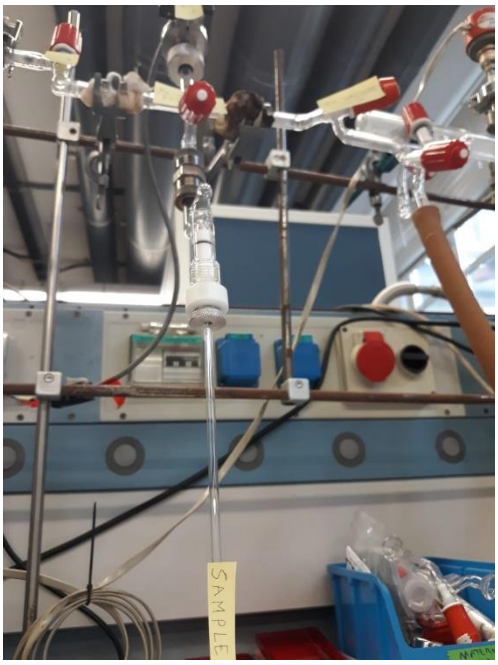

Figure SI2. Details of the connection to the NMR tube.

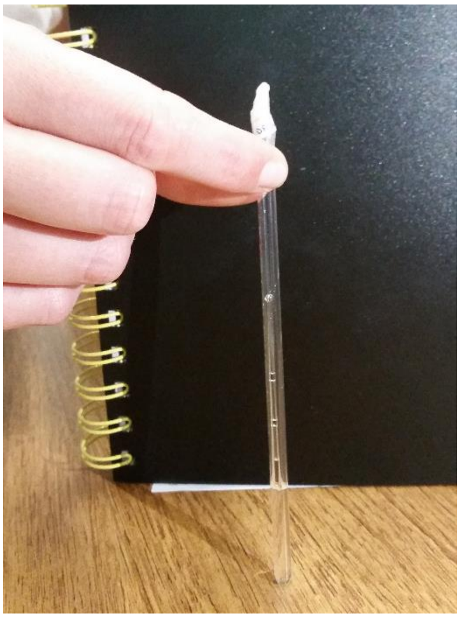

Figure SI3. Picture of the flame-sealed NMR tube.

NMR Data processing

The NMR experimental data were processed with the Bruker DOSY software to obtain the signal intensities at each applied gradient step. The normalized experimental signal decays *I(q, t)/I(0, t)* is plotted as function of q^2 (according to eq. 2) in a semi-logarithmic scale for the observation time used. The slope of the linear fit gives the msd value corresponding to the observed time t.

T_1 relaxation data were also calculated using a mono-exponential fitting according to the following equation:1

$$I(t) = I_0 e^{-t/T_1} \quad (1)$$

S.2 Molecular Dynamics Simulations

Technical details of the simulations: we have used the software package Gromacs[1] to run Molecular Dynamics (MD) simulations of several ILs systems. The Force Field used features the charge distribution developed by Canongia-Lopes and Padua (CL&AP FF)[2] while the internal parameters are based on the Amber[3] FF implementation in Gromacs. All bonds were constrained by the LINCS algorithm.[4] The leap-frog integrator was used with a time step of 1 fs and a cut-off of 10 Å for the van der Waals and short-range electrostatic interaction. The Particle-Mesh-Ewald (PME)[5] technique was used to handle long-range electrostatic interaction with an interpolation order of 4. Simulations were run in the NPT ensemble using the Berendsen thermostat[6] and the Parrinello-Rahman barostat[7,8] with applied isotropic periodic boundary conditions.

Force Field Validation: as mentioned above, the FF is slightly different, concerning the internal parameters for the cations, form that one used in Ref. [9], while the charge distribution is the same. Below we compare the radial distribution functions of Xe with several atoms of the ions obtained with the two versions of the FF, that one of Ref. [9] and that one used here for Xe@[C_4C_1im][Cl] and Xe@[C_4C_1im][PF_6]. Some minor technical details of the simulation set up, time length, number of particles in the box and software used are also changed. Nevertheless, the two sets of RDF are very close even though the RDF of Ref. [9] were obtained at 500 K, while these ones are obtained at 400 K.

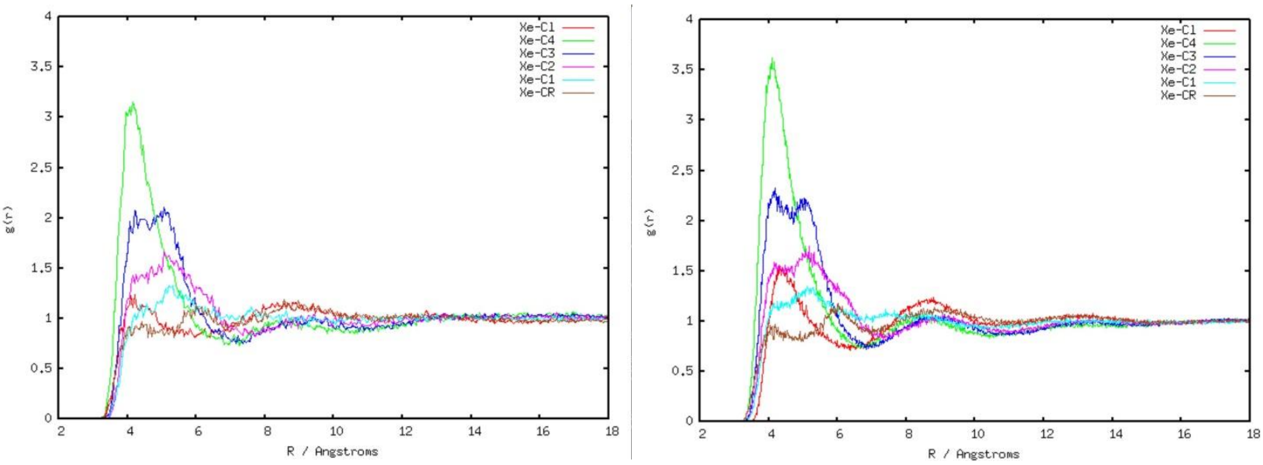

Figure SI4. RDF of the distance between Xe and the chloride anion, the carbon atoms of the alkyl chain from the terminal methyl C4 to C1, and with the C2 atom of the imidazolium ring, labeled CR. Left: from Ref. [9] at 500 K; right: this work at 400 K.

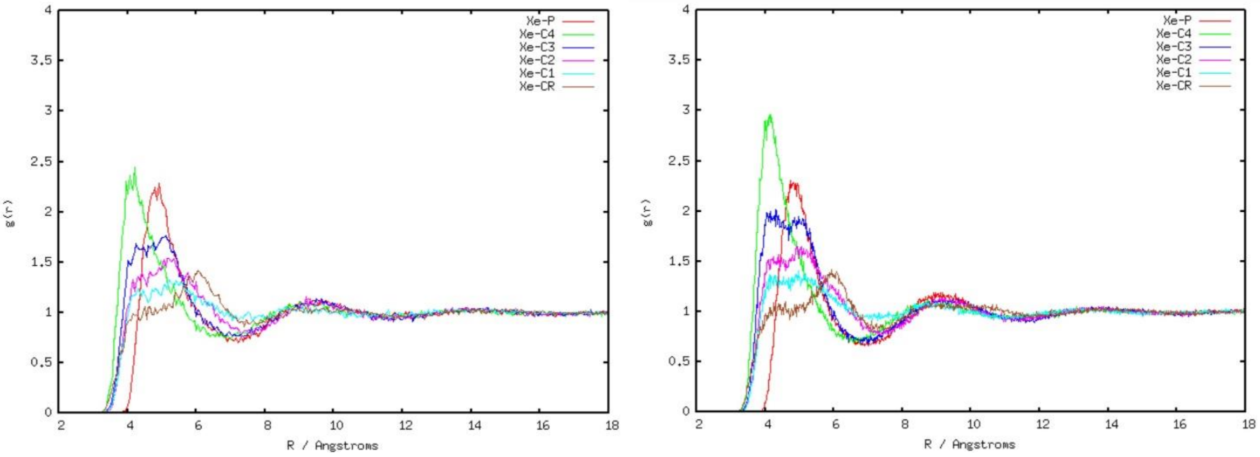

Figure SI5. RDF of the distance between Xe and the P of $[PF_6]$ anion, the carbon atoms of the alkyl chain from the terminal methyl C4 to C1, and with the C2 atom of the imidazolium ring, labeled CR. Left: from Ref. [9] at 500 K; right: this work at 400 K.

From Figures SI4 and SI5 it is clear that xenon, as already found in Ref. [9], is preferentially solvated by the alkyl chains. Moreover, only for the $[PF_6]$ salt, a significant interaction between xenon and the anion is observed.

To test whether the preferential solvation from the alkyl chains is due to an artefact of the interaction parameters between Xenon and the ions of the ILs, we also report here the interaction energy calculated with the present FF between Xenon and a single ion pair in two typical geometries: the first geometry, labeled Xe-CHAIN, features Xe at a variable distance from carbon 8 of $[C_{10}C_1im][Cl]$, measured perpendicularly to the all-trans alkyl chain. In the second geometry, labeled Xe-RING, the Xe atom is on top of the imidazolium ring of the $[C_{10}C_1im][Cl]$ ion pair at variable distance from the geometric center of the ring. The two geometries are shown in Figure SI6. The single $[C_{10}C_1im][Cl]$ ion has been optimized at the B3LYP-D3[10,11] level of theory. The total energy of the system is calculated by means of dispersion corrected functionals, B3LYP-D3[10,11] and M062X[12] with the 6-31+G** basis set for C, H, N, Cl and LANL2DZecp for Xenon. Basis sets were downloaded from the Basis set exchange website.[13]

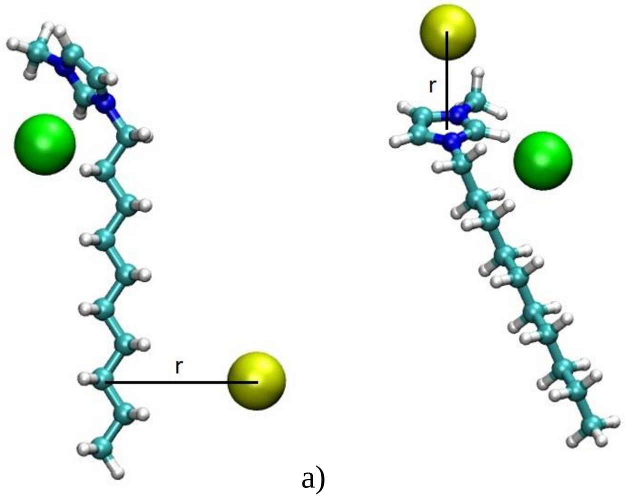

a) b)

Figure SI6. Model systems of $[C_{10}C_1im][Cl]$, a) Xe-CHAIN and b) Xe-RING, used to calculate the interaction energy of Xe (yellow) with either the alkyl chain or the imidazolium ring. In green the chloride anion.

In Figure SI7 we report the interaction energies of the two systems as a function of the Xe distance from carbon C8 of the alkyl chain and the geometric center of the imidazolium ring. The results obtained at the B3LYP-D3 show a very good agreement with the results obtained using the classical FF as far as the position of the minimum and contact distance is concerned. The value at the minimum is, however, calculated somewhat lower. With the M062X functional, the agreement for the Xe-CHAIN geometry is almost perfect while the Xe-RING geometry still exhibit some disagreement with the DFT results. It is clear that polarization effects, and possibly some induced charge transfer from Xe to the imidazolium ring, cannot be reproduced by the non-polarizable FF. However, the

important point is that both the classical FF and the dispersion corrected DFT results show that same relative weight of the interaction of Xe with the alkyl chains and the ring, the interaction with the imidazolium ring being the stronger one.

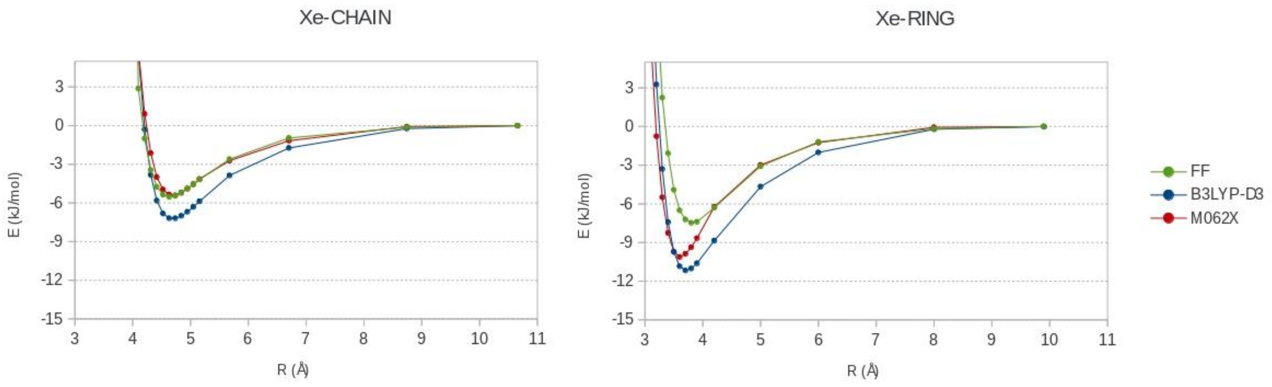

Figure SI7. Interaction energy at the classical FF level and DFT/6-31+G**(LANL2DZecp) levels of theory.

Simulation box properties: boxes were built starting from previous simulations[9,14] of the butyl systems and changing gradually the alkyl chain length after expanding the box to avoid overlap. Each system was then quickly relaxed to the volume under NPT conditions and equilibrated for 12 ns. The equilibration run was followed by a production run of 60 ns; configurations were saved every ps for further analysis. A first set of simulations was run with a box containing 500 ion pairs of [$C_nC_1$im][X] (n = 2, 4, 6, 8, 10 and X = Cl^-, PF_6^-) plus a Xe atom: for all systems these boxes were simulate at 350 K, 400 K, 450 K and 500 K and pressure of 1 bar. Inspection of the cation and anion diffusion coefficients revealed that some short-chain systems at the lower temperature were in a glassy state, rather than a liquid state. In a second set of simulations, the temperature was set to 400 K and the pressure to 1 bar. The boxes of this second set, contained 250 ion pairs plus a Xe atom. Three independent runs were produced for each system in order to estimate, together with the results of the first set of simulation at the same temperature of 400 K, the error associated with the diffusion coefficient of xenon. The systems studied by MD simulations are reported in Table S1. In Table S2 we report some additional data concerning the calculated and experimental densities. The densities of the simulated systems are in very good agreement with the available experimental data and estimates at 400 K.

Additional simulations were run for Xe@hexane and Xe@decane to estimate the diffusion of xenon in the liquid alkane. The FF parameters for decane were the same used for the hydrophobic part of the alkyl chain of decylimidazolium salts. Again, three independent box were generated containing 250 alkane molecules and one xenon atom. The boxes were equilibrated for 30 ns and the subsequent three consecutive production runs lasted 60 ns. Since for these systems there is not any significant effect of the electrostatic interaction, as for ILs, slowing down the dynamics, we run the simulations at 300 K.

Table S1. Systems investigated by MD simulation

System	N ion pairs	T	EQ ns	PROD ns	N Runs

Xe@[C_2C_1im][Cl]	500	300, 350, 400, 450, 500	30	60	1
Xe@[C_4C_1im][Cl]	500	350, 400, 450, 500	30	60	1
Xe@[C_6C_1im][Cl]	500	300, 350, 400, 450, 500	30	60	1
Xe@[C_8C_1im][Cl]	500	350, 400, 450, 500	30	60	1
Xe@[$C_{10}C_1$im][Cl]	500	350, 400, 450, 500	30	60	1
Xe@[C_2C_1im][Cl]	250	400	30	60	3
Xe@[C_4C_1im][Cl]	250	400	30	60	3
Xe@[C_6C_1im][Cl]	250	400	30	60	3
Xe@[C_8C_1im][Cl]	250	400	30	60	3
Xe@[$C_{10}C_1$im][Cl]	250	400	30	60	3
Xe@[C_2C_1im][PF_6]	500	350, 400, 450, 500	30	60	1
Xe@[C_4C_1im][PF_6]	500	350, 400, 450, 500	30	60	1
Xe@[C_6C_1im][PF_6]	500	350, 400, 450, 500	30	60	1
Xe@[C_8C_1im][PF_6]	500	350, 400, 450, 500	30	60	1
Xe@[$C_{10}C_1$im][PF_6]	500	350, 400, 450, 500	30	60	1
Xe@[C_2C_1im][PF_6]	250	400	30	60	3
Xe@[C_4C_1im][PF_6]	250	400	30	60	3
Xe@[C_6C_1im][PF_6]	250	400	30	60	3
Xe@[C_8C_1im][PF_6]	250	400	30	60	3
Xe@[$C_{10}C_1$im][PF_6]	250	400	30	60	3
Xe@hexane	250	300	30	60	3
Xe@decane	250	300	30	60	3

Table S2. Densities ρ, g/mL, of the systems studied: Experimental densities of the pure ILs at different temperatures (Exp. ρ). Estimated values for the pure ILs at 400 K obtained from the equation reported in Ref.[15] for the available compounds (Est. ρ). Simulated densities for the systems containing one Xe atom at 400 K (Sim. ρ). % deviation is calculated between the results of the simulations at 400 K and the estimated values at 400 K for the available compounds.

	Exp. ρ			Est. ρ	Sim. ρ	% deviation
	**303 K**	**333 K**	**373 K**	**400 K**	**400 K**	
Xe@[C_4C_1im][Cl]	1.079[a]				0.949	
Xe@[C_6C_1im][Cl]	1.035[a]		0.997		0.931	
Xe@[C_8C_1im][Cl]	1.007[a]	0.989[a]		0.949	0.920	-3.1

Xe@[$C_{10}C_1$im][Cl]	0.99				0.913	
Xe@[C_4C_1im][PF_6]	1.363	1.339		1.283	1.297	1.0
Xe@[C_6C_1im][PF_6]	1.288	1.264		1.212	1.239	2.2
Xe@[C_8C_1im][PF_6]	1.231	1.208		1.157	1.194	3.2
Xe@[$C_{10}C_1$im][PF_6]					1.159	

Calculation of the Mean Square Displacement (MSD): a representative example of the MSD obtained for [C_6C_1im][Cl] at T = 400 K from the three independent simulations using the 250 ion pairs box is reported in the Figures below.

The MSD is calculated by means of the corresponding routine of the Gromacs software package. The MSD is averaged over multiple time origins and over the number of equivalent particles. Since the boxes contain 250 ion pairs but only one Xe atom, the statistics on the Xe MSD degrades quickly with time, therefore the MSD of Xe obtained from independent simulation in the same conditions diverge before 10 ns. In contrast, for cation and anion, the MSD obtained by independent runs are nicely overlapped over a significant time range and clearly linear with time. For these reasons, the cation and anion D is obtained by fitting the MSD over a relatively long time range, up to 20 ns, while for Xe we limit the fitting to a 2 ns window. The final values of the diffusion coefficient, are then obtained as the average of the D obtained by fitting the MSD of the three independent runs at each state point.

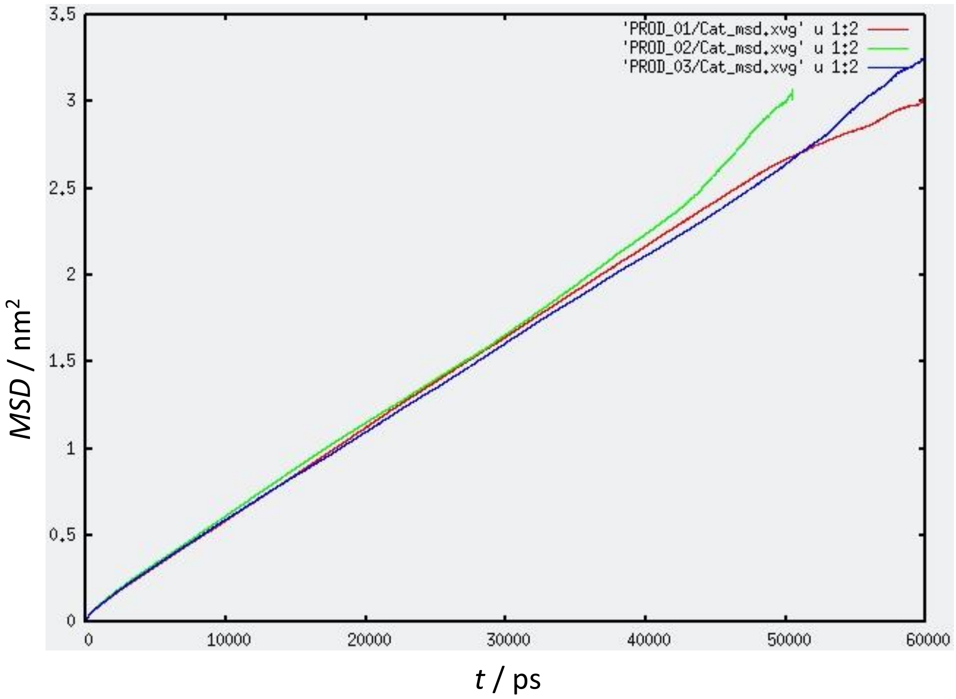

Figure SI8. MSD of the cation of [C_6C_1im][Cl] at 400 K from the three independent run of the 250 ion pairs box. *D* is calculated by fitting from 2000 to 20000 ps, D = 6·*MSD* ·*t*.

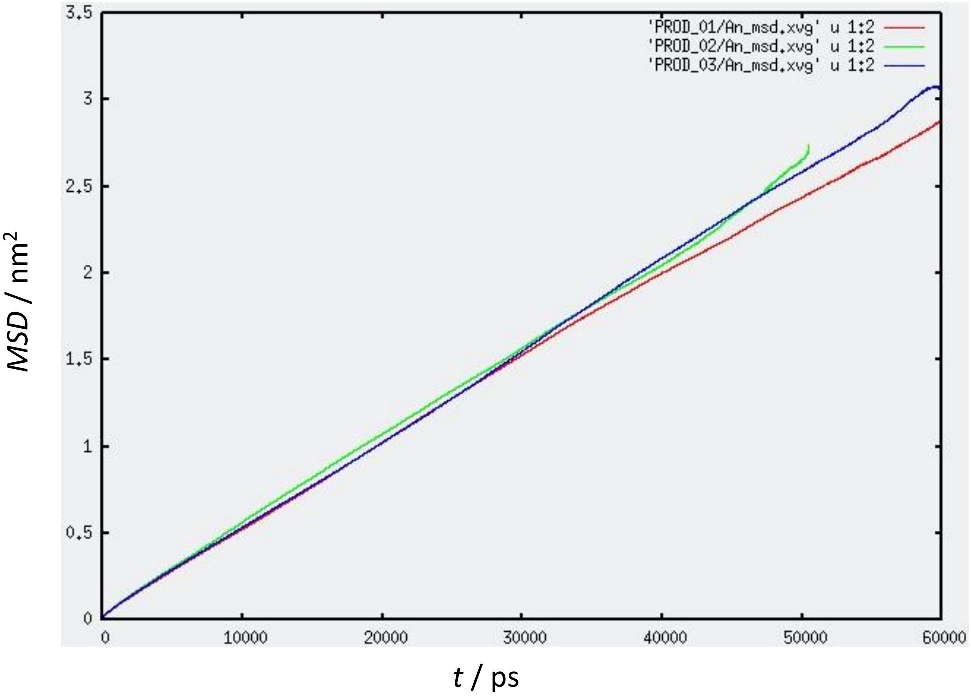

Figure SI9. MSD of the anion of [C_6C_1im][Cl] at 400 K from the three independent run of the 250 ion pairs box. *D* is calculated by linear fitting from 2000 to 20000 ps, D = 6·*MSD* ·*t*.

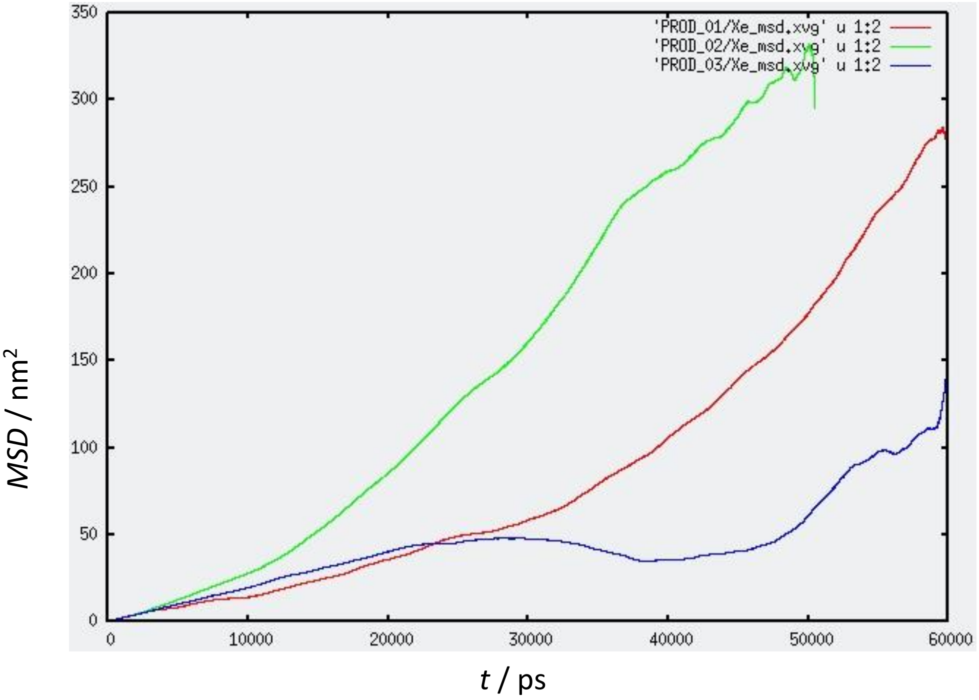

Figure SI10. MSD of xenon in [C_6C_1im][Cl] at 400 K from the three independent run of the 250 ion pairs box. *D* is calculated by linear fitting from 200 to 5000 ps, D = 6·*MSD* ·*t*.

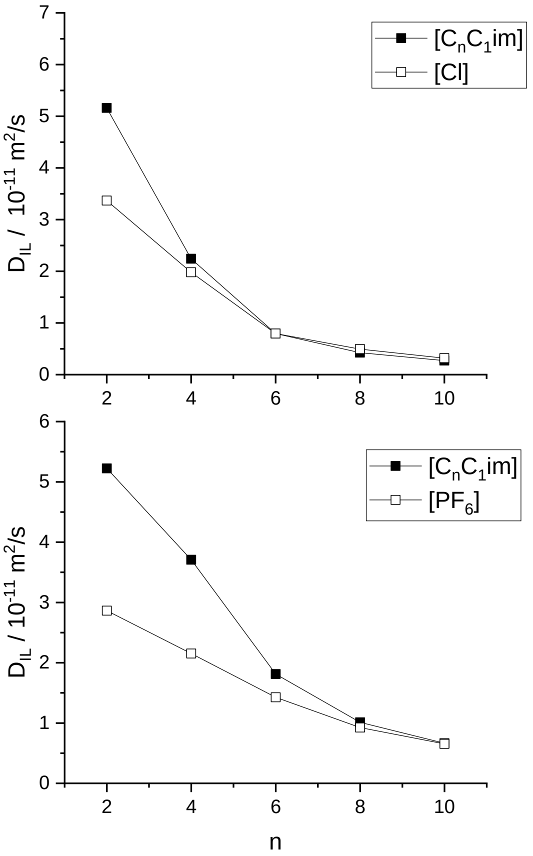

Figure SI11. Diffusion coefficients of ILs cation and anion obtained from the MD simulations at T = 400 K.

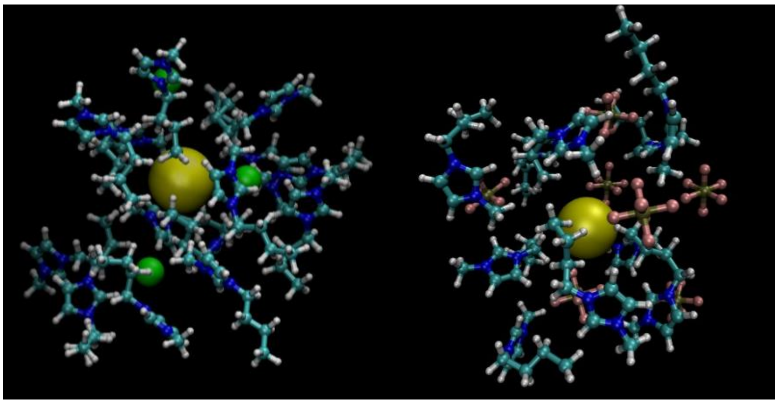

Figure SI12. Clusters of (left) Xe@[C_4C_1im][Cl] and (right) Xe@[C_4C_1im][PF_6] used to calculate the average ^{129}Xe chemical shift, see Ref. [9] The same average structure of the imidazolium salts is obtained in the present MD simulations.

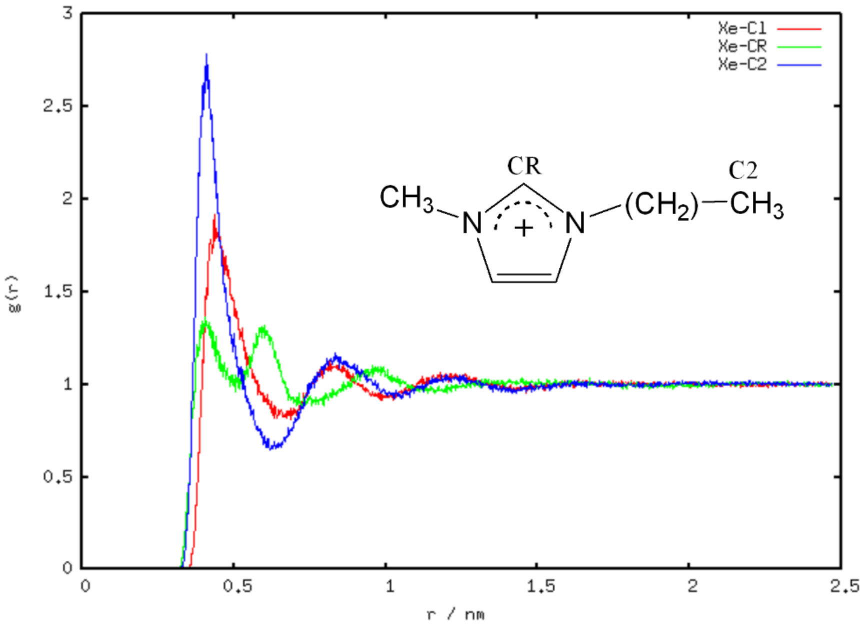

Figure SI13. RDF of xenon in Xe@[C_2C_1im][Cl] with chloride (red), imidazolium ring carbon CR and terminal alkyl carbon C2. T = 400 K.

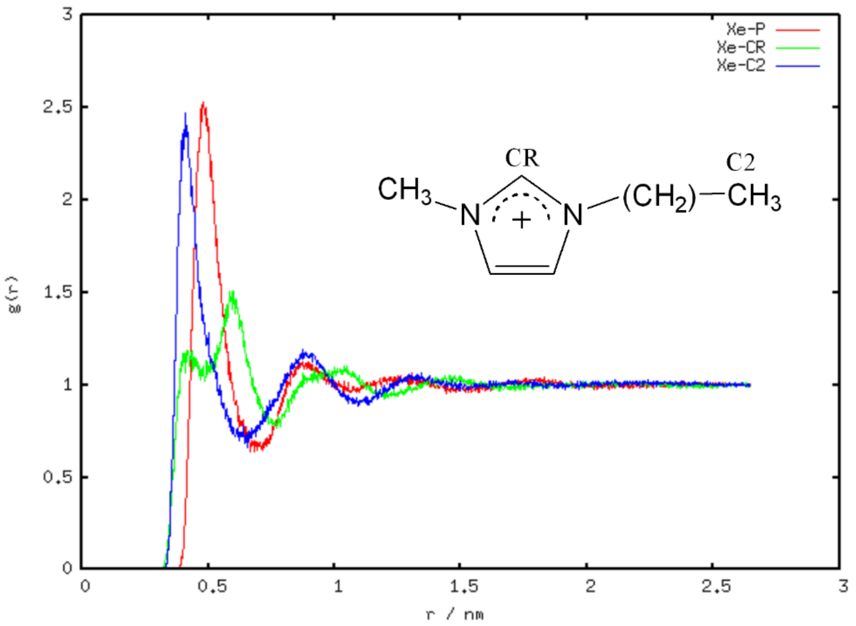

Figure SI14. RDF of xenon in Xe@[C_2C_1im][PF_6] with P of the anion (red), imidazolium ring carbon CR and terminal alkyl carbon C2. T = 400 K.

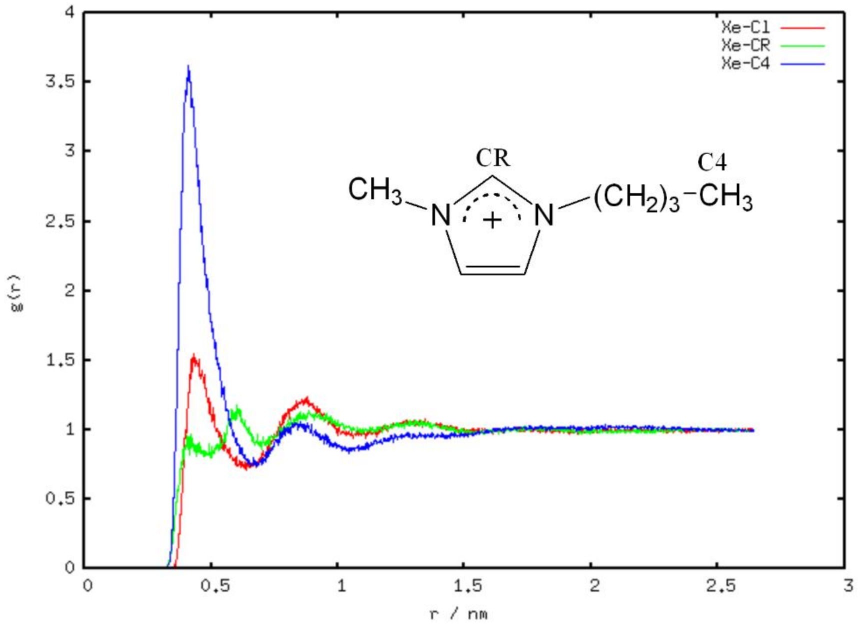

Figure SI15. RDF of xenon in Xe@[C_4C_1im][Cl] with chloride (red), imidazolium ring carbon CR and terminal alkyl carbon C4. T = 400 K.

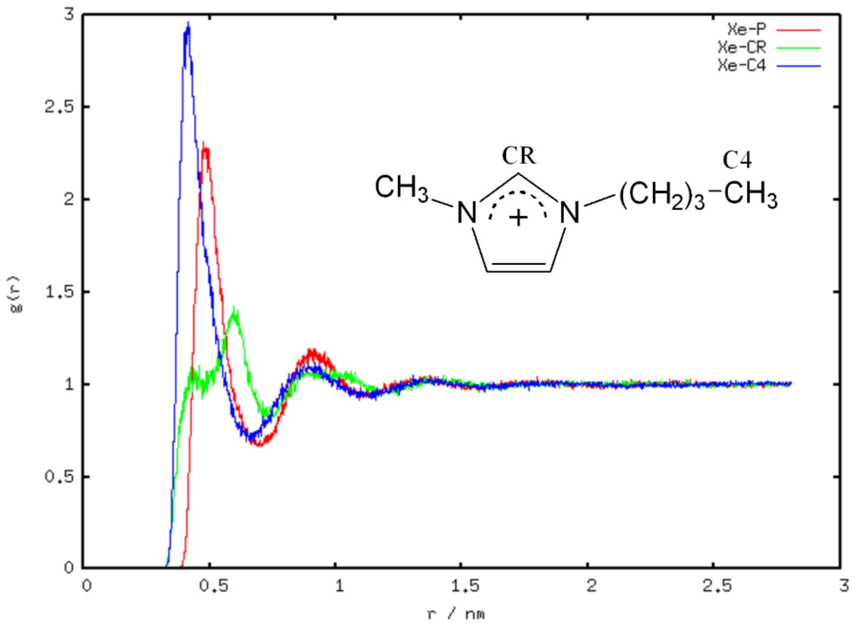

Figure SI16. RDF of xenon in Xe@[C_4C_1im][PF_6] with P of the anion (red), imidazolium ring carbon CR and terminal alkyl carbon C4. T = 400 K.

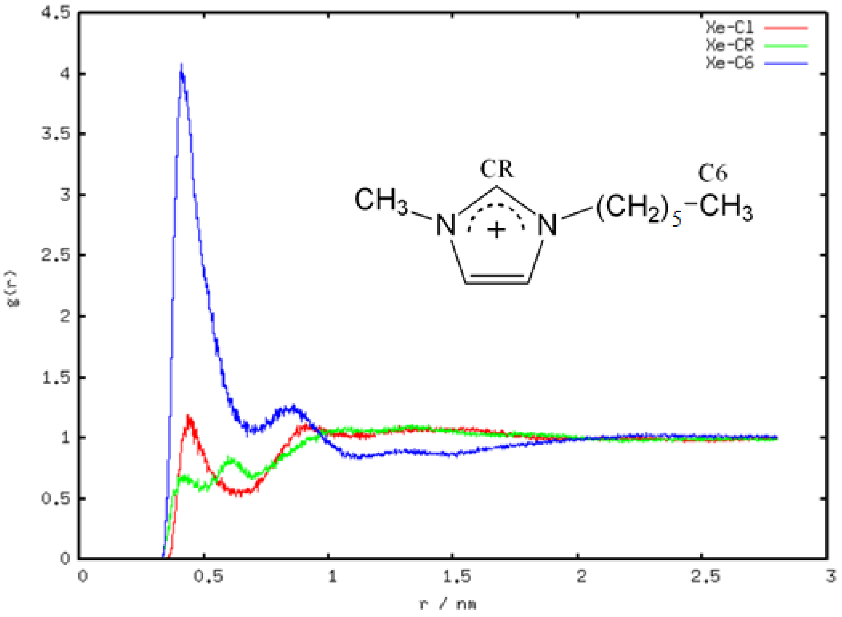

Figure SI17. RDF of xenon in Xe@[C_6C_1im][Cl] with chloride (red), imidazolium ring carbon CR and terminal alkyl carbon C6. T = 400 K.

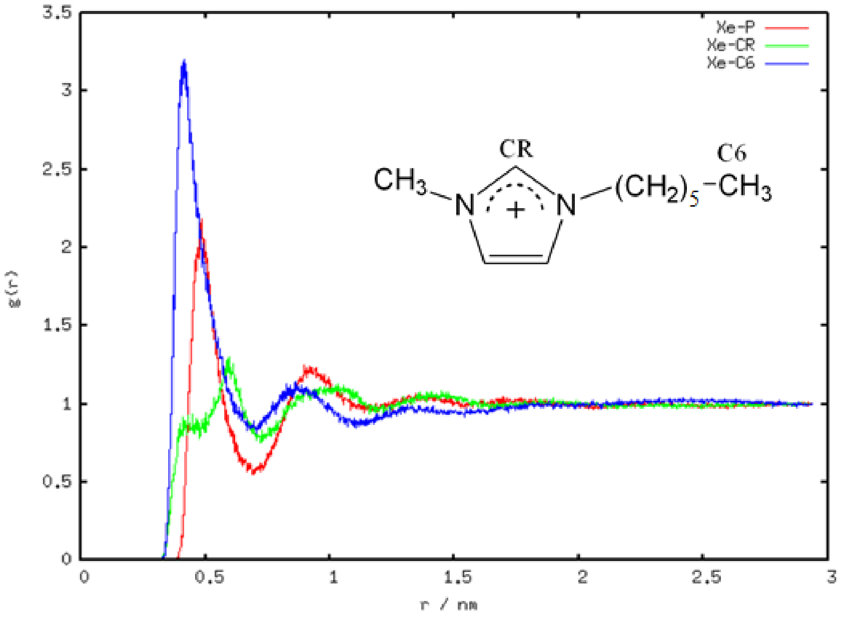

Figure SI18. RDF of xenon in Xe@[C_6C_1im][PF_6] with P of the anion (red), imidazolium ring carbon CR and terminal alkyl carbon C6. T = 400 K.

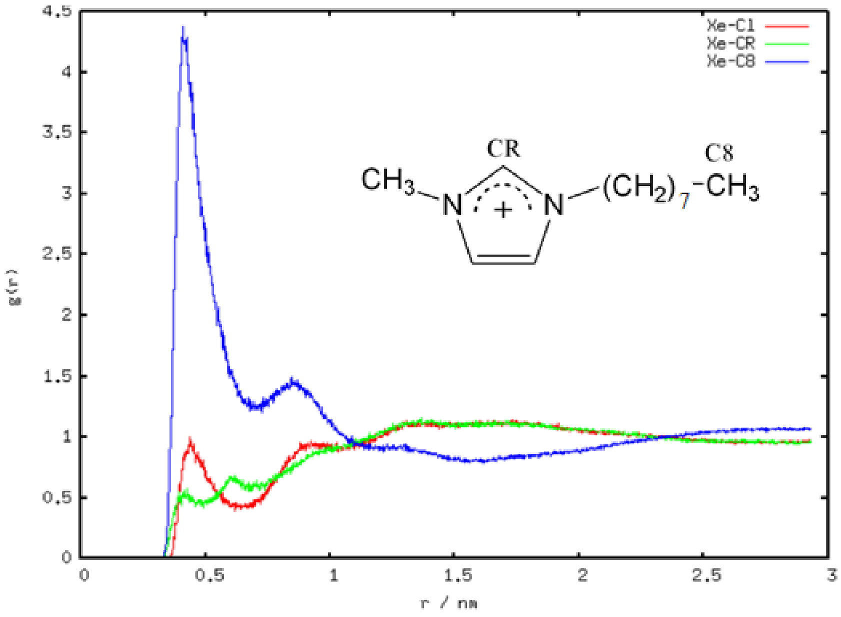

Figure SI19. RDF of xenon in Xe@[C_8C_1im][Cl] with chloride (red), imidazolium ring carbon CR and terminal alkyl carbon C8. T = 400 K.

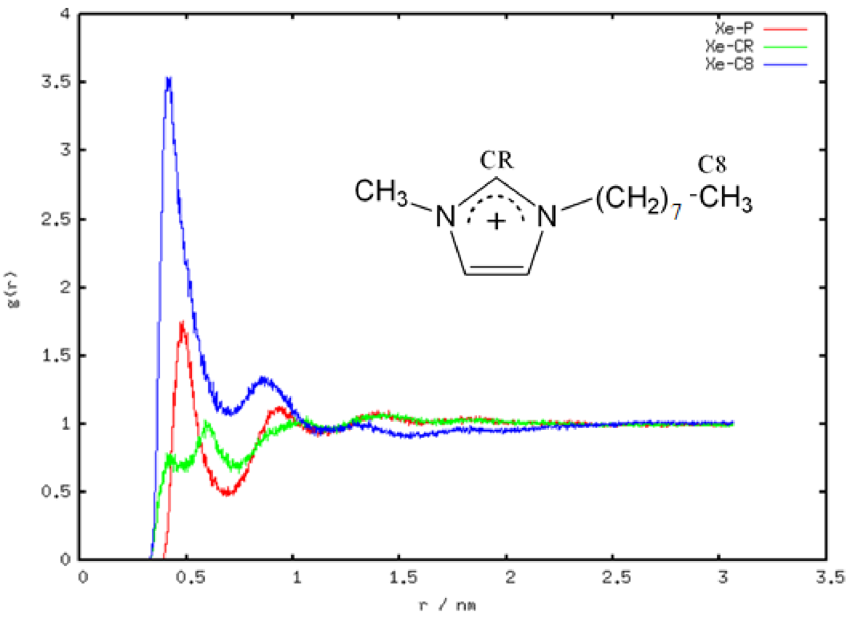

Figure SI20. RDF of xenon in Xe@[C_8C_1im][PF_6] with P of the anion (red), imidazolium ring carbon CR and terminal alkyl carbon C8. T = 400 K.

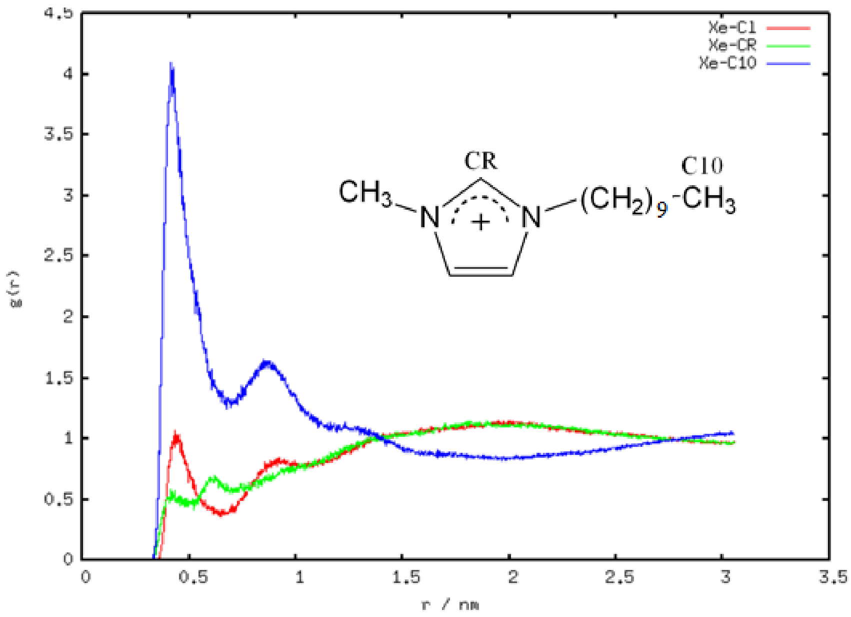

Figure SI21. RDF of xenon in Xe@[$C_{10}C_1$im][Cl] with chloride (red), imidazolium ring carbon CR and terminal alkyl carbon C10. T = 400 K.

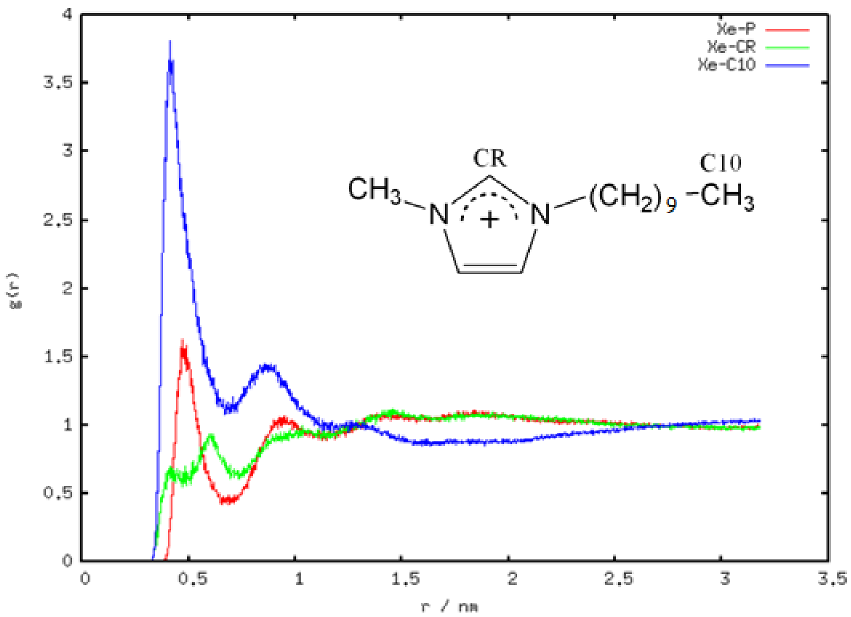

Figure SI22. RDF of xenon in Xe@[$C_{10}C_1$im][PF_6] with P of the anion (red), imidazolium ring carbon CR and terminal alkyl carbon C10. T = 400 K.

References

1 M. J. Abraham, T. Murtola, R. Schulz, S. Páll, J. C. Smith, B. Hess and E. Lindahl, *SoftwareX*, 2015, **1**–**2**, 19–25.

2 J. N. C. Lopes, J. Deschamps and A. A. H. A. H. Padua, *J. Phys. Chem. B*, 2004, **108**, 11250.

3 D. A. Case, I. Y. Ben-Shalom, S. R. Brozell, D. S. Cerutti, T. E. I. Cheatham, V. W. D. Cruzeiro, T. A. Darden, R. E. Duke, D. Ghoreishi, M. K. Gilson, H. Gohlke, A. W. Goetz, D. Greene, R. Harris, N. Homeyer, S. Izadi, A. Kovalenko, T. Kurtzman, T. S. Lee, S. LeGra, D. M. York and P. A. Kollman, 2018.

4 B. Hess, H. Bekker, H. J. C. Berendsen and J. G. E. M. Fraaije, *J. Comput. Chem.*, 1997, **18**, 1463–1472.

5 T. Darden, D. York and L. Pedersen, *J. Chem. Phys.*, 1993, **98**, 10089–10092.

6 H. J. C. Berendsen, J. P. M. Postma, W. F. van Gunsteren, A. DiNola and J. R. Haak, *J. Chem. Phys.*, 1984, **81**, 3684–3690.

7 M. Parrinello and A. Rahman, *J. Appl. Phys.*, 1981, **52**, 7182–7190.

8 S. Nosé and M. L. Klein, *Mol. Phys.*, 1983, **50**, 1055–1076.

9 G. Saielli, A. Bagno, F. Castiglione, R. Simonutti, M. Mauri and A. Mele, *J. Phys. Chem. B*, 2014, **118**, 13963–13968.

10 A. D. Becke, *J. Chem. Phys.*, 1993, **98**, 5648–5652.

11 S. Grimme, J. Antony, S. Ehrlich and H. Krieg, *J. Chem. Phys.*, 2010, **132**, 154104.

12 Y. Zhao and D. G. Truhlar, *Theor. Chem. Acc.*, 2008, **120**, 215–241.

13 K. L. Schuchardt, B. T. Didier, T. Elsethagen, L. Sun, V. Gurumoorthi, J. Chase, J. Li and T. L. Windus, *J. Chem. Inf. Model.*, 2007, **47**, 1045–1052.

14 D. Frezzato, A. Bagno, F. Castiglione, A. Mele and G. Saielli, *J. Mol. Liq.*, 2015, **210**, 272–278.

15 M. S. AlTuwaim, K. H. A. E. Alkhaldi, A. S. Al-Jimaz and A. A. Mohammad, *J. Chem. Eng. Data*, 2014, **59**, 1955–1963.
